# Investigating the amyloid–tau–neurodegeneration framework in Alzheimer's disease using semi‐supervised multimodal imaging data fusion

**DOI:** 10.1002/dad2.70360

**Published:** 2026-05-21

**Authors:** You Cheng, Adrián Medina, Cole Korponay, Christian F. Beckmann, David Harper, Lisa Nickerson, for the Alzheimer's Disease Neuroimaging Initiative

**Affiliations:** ^1^ McLean Hospital Belmont Massachusetts USA; ^2^ Mass General Brigham Boston Massachusetts USA; ^3^ Department of Psychiatry Harvard Medical School Boston Massachusetts USA; ^4^ Donders Institute for Brain Cognition and Behaviour Department of Medical Neuroscience Radboud University Medical Centre Nijmegen the Netherlands; ^5^ Centre for Functional MRI of the Brain (FMRIB) Nuffield Department of Clinical Neurosciences Wellcome Centre for Integrative Neuroimaging University of Oxford Oxford UK

**Keywords:** Alzheimer's disease, amyloid–tau–neurodegeneration framework, latent components, multimodal data fusion, multimodal neuroimaging, semi‐supervised learning

## Abstract

**INTRODUCTION:**

Alzheimer's disease (AD) heterogeneity complicates diagnosis and prognosis. Uncovering amyloid–tau–neurodegeneration (A–T–N) patterns may improve diagnostic prediction.

**METHODS:**

We applied SuperBigFLICA (SBF), a semi‐supervised multimodal fusion method, to gray matter density, cortical thickness (CT), pial surface area, amyloid and tau positron emission tomography maps from 274 Alzheimer's Disease Neuroimaging Initiative 3 participants to derive 50 latent components predictive of cognitive decline. Subject loadings were then used to predict diagnosis (cognitively normal, mild cognitive impairment, dementia) and apolipoprotein E (*APOE*) ε4 status via least absolute shrinkage and selection operator logistic regression, compared to demographic, single‐modality, and naïve fusion comparator models.

**RESULTS:**

SBF modestly predicted out‐of‐sample concurrent clinical severity (Clinical Dementia Rating Sum of Boxes; *r* = 0.21), yet models using SBF‐derived loadings were among the strongest comparator models (area under the receiver operating characteristic curve; = 0.80 for diagnosis; 0.83 for *APOE* ε4). Amyloid alterations in sensory areas best separated dementia, while a tri‐modal tau–neurodegeneration pattern related to disease progression. Loadings were validated through cerebrospinal fluid correlations.

**DISCUSSION:**

SBF improves prediction and reveals interpretable patterns that better classify clinical diagnoses and *APOE* ε4 than traditional approaches.

## Highlights

- Semi‐supervised multimodal data fusion (SuperBigFLICA) integrates amyloid–tau–neurodegeneration (A–T–N) imaging patterns.
- Latent components successfully predict cognitive decline, clinical diagnosis, and apolipoprotein E (*APOE*) ε4 status.
- A joint tau–neurodegeneration brain pattern tracked the full disease progression.
- Amyloid alterations in sensory areas were associated with *APOE* ε4 carrier status.
- Among the strongest models, SuperBigFLICA provides an interpretable linear framework for Alzheimer's disease and related dementias research.

## 1 INTRODUCTION

Alzheimer's disease (AD) is the most common cause of dementia, representing ≈ 60% to 80% of dementia cases and affecting nearly 7.2 million US adults aged ≥ 65 as of 2025.^1^ Clinically, AD is characterized by a progression of changes with aging that begin from cognitively normal (CN) status through mild cognitive impairment (MCI) to AD dementia. Although AD neuropathological change is defined by the presence of amyloid beta (Aβ) and tau pathology,^2^ individuals who meet these criteria often show substantial variability in the spatial distribution and relative burden of these pathologies across the brain. Further, significant variability in clinical severity across these stages complicates early diagnosis^3^ and underscores the need for methods that capture the multivariate nature of disease progression.

Multimodal neuroimaging provides a rich and comprehensive view of brain changes in AD, as different imaging modalities are sensitive to both overlapping and distinct neurobiological processes. For example, structural magnetic resonance imaging (MRI) provides a view of neurodegeneration, positron emission tomography (PET) of molecular pathology, and functional MRI (fMRI) of brain circuitry integrity. Structural MRI and PET provide complementary information for clinical classification in AD.^4^, ^5^ In addition to disease signals that are modality specific, some neurodegenerative processes are convergent across modalities, with changes in the underlying processes manifesting across multiple modalities. For example, brain atrophy observed on MRI is highly correlated with tau pathology detected via tau PET, particularly in medial temporal regions.^6^ Capturing both modality‑unique and shared signals is therefore essential for a more comprehensive mechanistic model. Furthermore, AD pathology manifests spatially distinct patterns across modalities. For example, high early amyloid burden but minimal atrophy may be observed in regions such as the frontal cortex; conversely, significant hippocampal atrophy may be observed with relatively low local amyloid deposition.^7^

Despite advances in machine learning models for predicting clinical diagnoses or AD risk factors (e.g., apolipoprotein E [*APOE*] ε4 carrier status), existing approaches predominantly focus on categorical predictions (e.g., MCI to AD conversion) or rely on limited non‐imaging and/or imaging modalities (e.g., only neuropsychological tests,^8^, ^9^, ^10^, ^11^ diffusion tensor imaging [DTI],^9^ MRI,^10^, ^11^, ^12^, ^13^, ^14^, ^15^, ^16^ fMRI, ^15^, ^17^ electroencephalogram [EEG]).^18^ These models typically lack interpretability regarding how features across modalities collectively relate to the target variable, limiting insight into underlying disease mechanisms. Moreover, traditional pairwise modality comparisons, such as voxel‐based analyses between fluorodeoxyglucose (FDG) PET hypometabolism and MRI atrophy, cannot scale to incorporate broader multimodal data.^19^ This represents a critical gap in the literature, as leveraging shared variance across imaging modalities toward target variable prediction may yield more biologically meaningful early detection of heterogenous disease.

Addressing these limitations, recent advances in multimodal neuroimaging fusion enable the identification of multimodal brain patterns via joint analysis that effectively increases both the power of detecting convergent effects and the interpretability of the findings.^20^ These patterns are typically linked post hoc to behavioral, clinical, and demographic phenotypes.^21^, ^22^, ^23^ In this study, we apply SuperBigFLICA (SBF), a framework evolved from FMRIB's Linked Independent Component Analysis (FLICA)—a multimodal Bayesian independent component analysis method for data fusion—that has been scaled for big data and adapted for simultaneous supervised learning of a target variable(s).^24^ SBF is a cutting‐edge data fusion approach that capitalizes on the benefits of multiple modalities being measured within each subject while modeling shared covariance across imaging modalities to identify linked multimodal latent spatial components that are maximally predictive of one or more continuous target variable(s). This simultaneous unsupervised multimodal decomposition with supervised target prediction enables both robust prediction and insights into the brain spatial patterns most associated with these outcomes. This approach bridges the gap between prediction and neurobiological understanding and increases sensitivity to gradual disease progression and individual variability, allowing subtler brain–behavior relationships to emerge.

To demonstrate the utility of SBF for multimodal imaging in AD, we trained the model on cognitive decline (Clinical Dementia Rating Sum of Boxes [CDR‐SOB]), a continuous measure of cognitive status that is closely aligned with clinical diagnosis, using it as a proxy training variable. We hypothesized that (1) cross‐modal covariance patterns spanning amyloid, tau, and neurodegeneration (A–T–N) modalities would capture variance relevant to clinical outcomes and improve prediction of clinical diagnoses, and (2) latent loadings derived from these patterns could be further leveraged to enhance prediction of genetic risk, specifically *APOE* ε4 carrier status.

**RESEARCH IN CONTEXT**

1. **Systematic review**: We searched PubMed and Google Scholar for studies applying multimodal neuroimaging fusion and explainable machine learning in Alzheimer's disease (AD). Prior work has largely focused on single‐modality analyses or black‐box prediction models, with limited integration across amyloid, tau, and neurodegeneration biomarkers.
2. **Interpretation**: Using SuperBigFLICA (SBF), a semi‐supervised multimodal fusion framework, we show that training on a continuous variable related to cognitive decline yields latent component loadings that can be leveraged to predict clinical diagnoses and *apolipoprotein E* (*APOE* ε4) carrier status. SBF‐derived loadings were among the strongest performers across comparator models based on single‐modality features or naïve fusion, while also providing interpretable multimodal brain patterns linked to disease processes.
3. **Future directions**: Broader adoption of semi‐supervised multimodal fusion could improve prediction and mechanistic understanding in AD and related dementias. Future studies should extend this framework to different disease risk factors and additional modalities.

## 2 METHODS

### 2.1 Study participants

Data were obtained from the Alzheimer's Disease Neuroimaging Initiative 3 (ADNI‑3).^25^ We selected one visit per participant that contained complete data for (1) Aβ PET; (2) tau PET; (3) 3T T1‑weighted MRI; (4) demographics (age, sex); and (5) CDR‐SOB,^26^ which sums six domains (memory, orientation, judgment/problem solving, community affairs, home/hobbies, personal care) each scored 0 to 3 for a total of 0 to 18. One proposed translation^27^ interprets scores of 0 as CN, 0.5 to 4 as MCI/subjective cognitive decline (SCD), 4.5 to 9 as mild dementia, 9.5 to 15.5 as moderate dementia, and 16 to 18 as severe dementia. The resulting cohort spanned CN, MCI, and AD diagnoses; amyloid status was not used as an inclusion criterion so that the sample would represent cognitive aging broadly rather than the biomarker‑defined AD continuum.

Please refer to Material SA.1 in supporting information for ADNI eligibility criteria and Material SA.2–3 for image acquisition and preprocessing details.

### 2.2 SBF model

**FIGURE 1:**
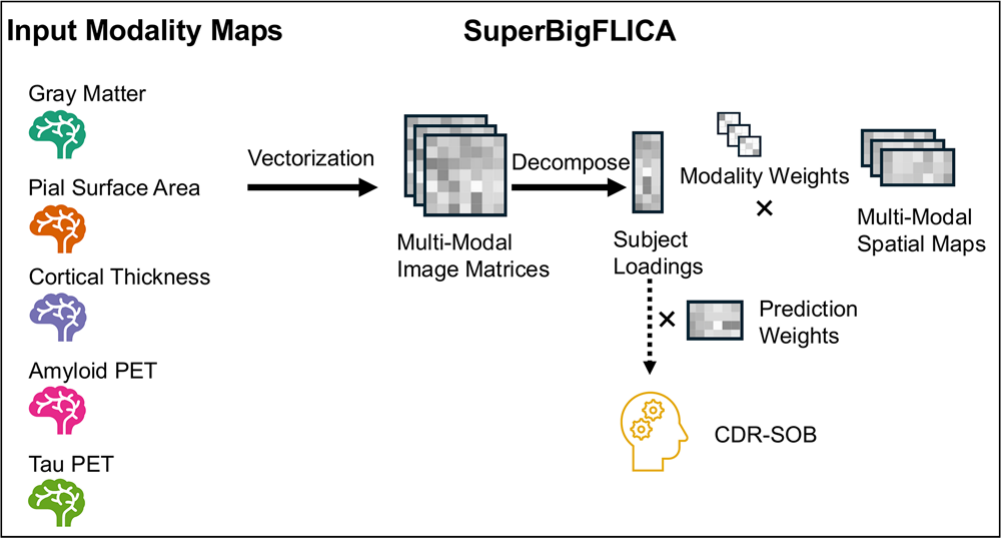
Implementation of the SuperBigFLICA approach for semi‐supervised multimodal fusion and phenotype discovery. Workflow of the SuperBigFLICA approach in this study. Inputs include five imaging modality maps from the ADNI‐3 dataset: gray matter density (GM), cortical thickness (CT), pial surface area (PSA), amyloid PET Centiloid (AMY), and tau PET SUVR (TAU). These maps, together with the continuous target (i.e., CDR‐SOB scores), were fed into the SuperBigFLICA algorithm for multimodal learning. The outputs are the subject loadings, multimodal spatial maps and their prediction weights for maximal prediction of the target variable. ADNI, Alzheimer's Disease Neuroimaging Initiative; CDR‐SOB, Clinical Dementia Rating Sum of Boxes; ICA, independent component analysis; PET, positron emission tomography; SUVR, standardized uptake value ratio.

We used SBF,^24^ a semi‐supervised multimodal fusion framework, to jointly decompose neuroimaging data and predict clinical decline (see Material SB in supporting information for SBF details and GitHub repository^28^ for code). Five voxel‐/vertex‐wise maps per participant (gray matter density [GM], cortical thickness [CT], pial surface area [PSA], amyloid PET Centiloid [AMY], tau PET standardized uptake value ratio [TAU]) were each concatenated across subjects to create five modality series that were fed into SBF to decompose the data into 50 latent components that were maximally predictive of CDR‐SOB (see Figure 1). We estimated 50 latent components based on prior experience with independent component analysis (ICA) for resting‐state fMRI and linked ICA for multimodal data fusion, at which dimensionality the spatial maps showed patterns consistent with network and sub‐network structures observable at model orders between ≈ 30 to 70 components.^29^ We also conducted sensitivity analyses across nine dimensionality configurations (10–50 components) and five random initialization rounds to confirm the model stability. For SBF's embedded cross‐validation, data were randomly partitioned into training (70%), validation (15%), and test (15%) sets, with all participants from a given imaging site assigned to the same split to prevent site‑related data leakage. The analyses were conducted in Python 3.9 using PyTorch 2.1.2.

The model produced (1) participant‐level latent loadings for both training and test sets, (2) spatial maps for each of the 50 components, and (3) outcome predictions evaluated by the correlation between predicted and observed scores in the independent test set. The output spatial maps were thresholded individually using mixture model–based thresholding in FSL MELODIC (mmthresh = 0.5), which explicitly models the distributions of the noise and signal classes in each map. Scanner manufacturer effects were removed from all model loadings (SBF, ICA, and principal component analysis [PCA]) via ComBat harmonization^30^, ^31^, ^32^ (full details in Material SC.1–6, Tables S1–S11, and Figures S1–S2 in supporting information), and all subsequent classification analyses were conducted on the harmonized loadings.

### 2.3 Prediction of clinical diagnoses and *APOE* ε4 carrier status

The SBF model is designed to identify latent representations that generalize across related outcomes;^24^ therefore, we tested whether subject loadings derived from the SBF model trained to predict CDR‐SOB could transfer to clinical diagnoses and *APOE* ε4 carrier status. Specifically, binary least absolute shrinkage and selection operator (LASSO) logistic regression models were trained to classify both clinical diagnosis (CN, MCI, dementia) and *APOE* ε4 carrier status. The primary input feature set comprised latent loadings from all 50 SBF components. LASSO was chosen to prevent multicollinearity while enabling feature selection. Model fitting used the latent loadings from the SBF training set, and evaluation used the latent loadings from the SBF test set. See Material SD in supporting information for classification model details. Prediction performance of SBF loadings was compared against additional comparator model feature sets serving as reference points to assess whether SBF adds value over single‐modality or naïve fusion approaches (detailed in Material SE in supporting information).

Additionally, loadings from the components most predictive of diagnoses and *APOE* ε4 were correlated with cerebrospinal fluid (CSF) amyloid (Aβ42) and tau (phosphorylated tau [p‐tau]181) in the subset of participants with available CSF data to help interpret and validate their neuropathological associations—including for components derived from modalities in which links to amyloid and tau are not direct. Only CSF samples collected within 1 year of the imaging visit were included, and when multiple samples were available, the draw closest to the imaging date was chosen.

## 3 RESULTS

### 3.1 Study participants

The ADNI‐3 sample included 274 participants with all required data split into training (*n* = 192), validation (*n* = 41), and test (*n* = 41) sets. See Table 1 and Material SF in supporting information for details.

**TABLE 1:** Summary statistics of the study population.

Characteristics	Total (*n* = 274)	Missing, N (%)	Train (*n* = 192; 70%)	Validation (*n* = 41; 15%)	Test (*n* = 41; 15%)	Statistical test (*p* value)
**Age, mean (SD), years**	70.8 (6.9)		70.5 (6.8)	71.1 (7.2)	71.8 (6.6)	0.529
**Sex, N (%)**						0.119
Male	120 (43.8)		79 (41.1)	24 (58.5)	17 (41.5)	
Female	154 (56.2)		113 (58.9)	17 (41.5)	24 (58.5)	
**Race, N (%)**		1 (0.4)	1 (0.5)	0 (0.0)	0 (0.0)	0.893
White	254 (92.7)		176 (91.7)	40 (97.6)	38 (92.7)	
Black or African American	9 (3.3)		6 (3.1)	1 (2.4)	2 (4.9)	
AI or AN	1 (0.4)		1 (0.5)	0 (0.0)	0 (0.0)	
Asian	4 (1.5)		4 (2.1)	0 (0.0)	0 (0.0)	
More than one race	5 (1.8)		4 (2.1)	0 (0.0)	1 (2.4)	
**Ethnicity, N (%)**		1 (0.4)	1 (0.5)	0 (0.0)	0 (0.0)	0.841
Hispanic or Latino	10 (3.6)		7 (3.6)	2 (4.9)	1 (2.4)	
Not Hispanic or Latino	263 (96.0)		184 (95.8)	39 (95.1)	40 (97.6)	
**Education, N (%)**						0.594
High school or less	27 (9.9)		19 (9.9)	3 (7.3)	5 (12.2)	
Some college	40 (14.6)		33 (17.2)	4 (9.8)	3 (7.3)	
College	86 (31.4)		60 (31.2)	14 (34.1)	12 (29.3)	
Graduate	121 (44.2)		80 (41.7)	20 (48.8)	21 (51.2)	
**CDR‐SOB, N (%)**						0.695
0	163 (59.5)		116 (60.4)	22 (53.7)	25 (61.0)	
0.5–4	95 (34.7)		63 (32.8)	17 (41.5)	15 (36.6)	
4.5–9	16 (5.8)		13 (6.8)	2 (4.9)	1 (2.4)	
**MMSE, median (IQR)**	29.00 [27.00, 30.00]		29.00 [27.00, 30.00]	29.00 [27.00, 30.00]	29.00 [27.00, 30.00]	0.753
**MoCA, median (IQR)**	25.00 [22.00, 27.00]		25.00 [23.00, 28.00]	23.00 [20.00, 26.00]	25.00 [22.00, 27.00]	0.05
**ADAS‐COG, median (IQR)**		5 (1.8)	2 (1)	1 (2)	2 (4.8)	0.828
	14.00 [11.00, 20.00]		14.00 [10.25, 19.00]	15.00 [11.00, 23.25]	15.00 [11.00, 19.50]	
**NPI, median (IQR)**	0.00 [0.00, 3.00]		0.00 [0.00, 3.00]	0.00 [0.00, 4.00]	0.00 [0.00, 2.00]	0.248
**GDS, median (IQR)**	1.00 [0.00, 2.00]		1.00 [0.00, 2.00]	0.00 [0.00, 2.00]	1.00 [0.00, 1.00]	0.864
**Diagnosis, N (%)**						0.277
CN	175 (63.9)		127 (66.1)	22 (53.7)	26 (63.4)	
MCI	60 (21.9)		37 (19.3)	11 (26.8)	12 (29.3)	
Dementia	39 (14.2)		28 (14.6)	8 (19.5)	3 (7.3)	
***APOE* genotype**		2 (0.7)	2 (1.0)	0 (0.0)	0 (0.0)	0.732
*APOE* ε4 carrier	111 (40.5)		76 (39.6)	16 (39.0)	19 (46.3)	
*APOE* ε4 non‐carrier	161 (58.8)		114 (59.4)	25 (61.0)	22 (53.7)	
**CSF** Aβ**42, median (IQR), pg/mL**		62 (22.6)	44 (22.9)	9 (22.0)	9 (22.0)	
	1009.00 [689.77, 1545.75]		1009.00 [711.80, 1551.75]	872.80 [685.10, 1532.00]	1130.50 [622.12, 1395.50]	0.713
**CSF p‐tau 181, median (IQR), pg/mL**		63 (23.0)	43 (22.4)	10 (24.4)	10 (24.4)	
	20.17 [14.86, 28.83]		19.94 [14.74, 28.81]	20.64 [17.44, 30.53]	20.42 [14.71, 25.96]	0.614

*Notes*: Statistical analyses were performed using one‐way analysis of variance for normally distributed continuous variables, Kruskal–Wallis rank‐sum tests for continuous variables that did not meet normality assumptions, and chi‐squared tests for categorical variables. Normality was assessed using skewness and kurtosis (|skewness| > 1 or |kurtosis| > 3 was considered non‐normal). *APOE* ε4 prevalence was balanced across splits (χ^2^ *p* = 0.732) and across imaging sites (Fisher exact test with Monte Carlo simulation, *p* = 0.133). ADAS‐COG range = 0–70 (higher scores indicate greater impairment); CDR‐SOB range = 0–18 (higher scores indicate greater cognitive decline); GDS range = 0–15 (higher scores indicate more depressive symptoms); MMSE range = 0–30 (higher scores indicate better cognition); MoCA range = 0–30 (higher scores indicate better cognition); NPI range =  0–144 (higher scores indicate more severe neuropsychiatric symptoms).

Abbreviations: Aβ, amyloid beta; ADAS‐COG, Alzheimer's Disease Assessment Scale–Cognitive Subscale; AI/AN, American Indian/Alaska Native; *APOE*, apolipoprotein E; CN, cognitively normal; CDR‐SOB, Clinical Dementia Rating–Sum of Boxes; CSF, cerebrospinal fluid; GDS, Geriatric Depression Scale; IQR, interquartile range; MCI, mild cognitive impairment; MMSE, Mini‐Mental State Examination; MoCA, Montreal Cognitive Assessment; NPI, Neuropsychiatric Inventory; p‐tau, phosphorylated tau; SD, standard deviation.

### 3.2 Multimodal components demonstrate model robustness

**FIGURE 2:**
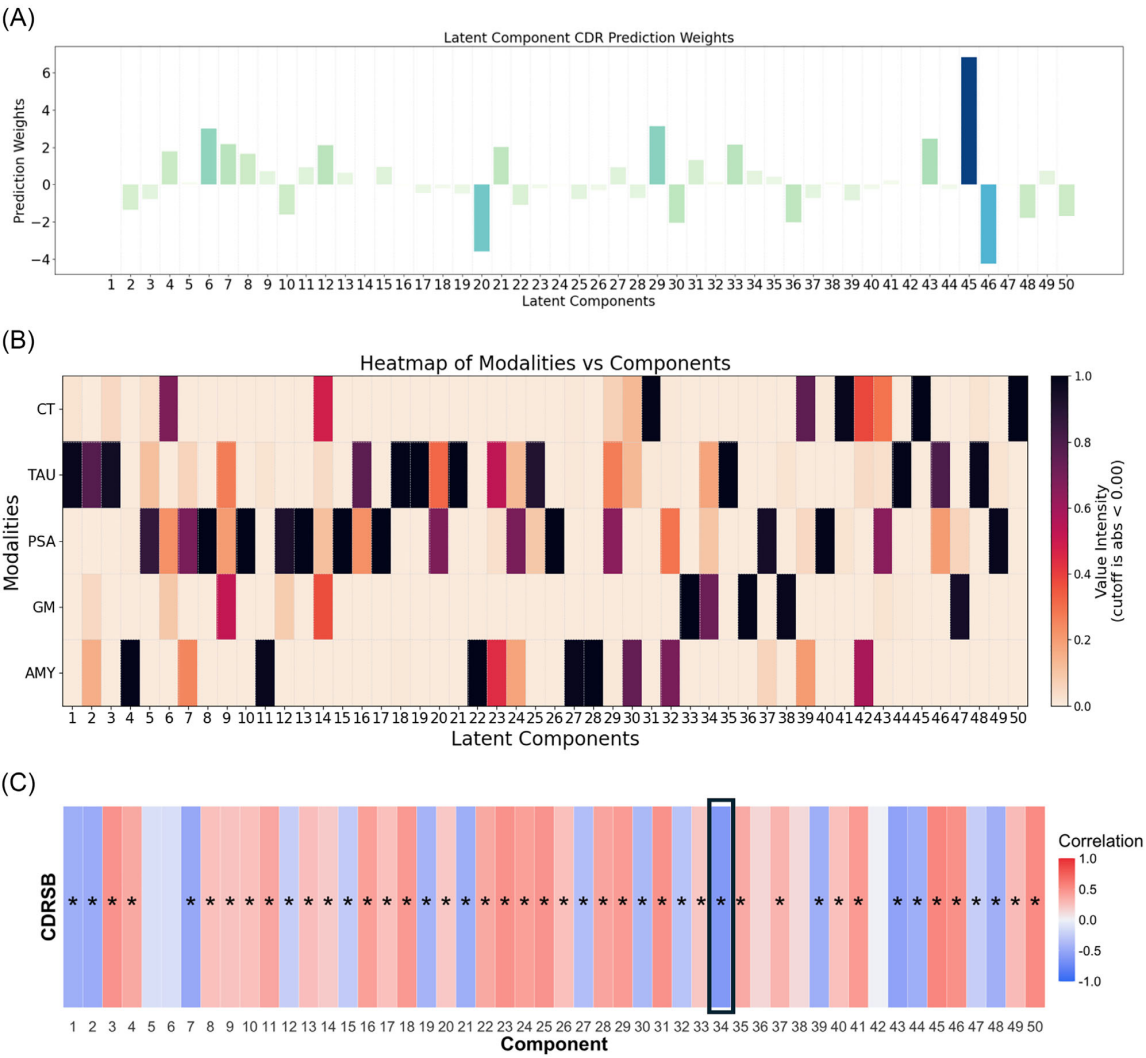
Weight distributions and component–CDR associations from SuperBigFLICA. A, Linear regression coefficients from the multivariable model predicting continuous CDR‐SOB across the 50 latent components. Coefficient magnitude reflects contribution to the global continuous outcome model, not task‐specific discriminative importance in downstream classification analyses. B, Modality weight distribution (unthresholded) showing the dominant modalities of each uni‐ or multi‐modal component. C, Pearson correlations between post‐ComBat component loadings and CDR‐SOB show nearly all components were linked to CDR‐SOB. Component 34 (emphasized with a black box) had the strongest correlation (*r* = −0.614, *p <* .001). Asterisks denote significance after Benjamini–Hochberg (BH) correction. Values indicate correlation coefficients; red reflects positive correlations; blue reflects negative correlations. CDR‐SOB, Clinical Dementia Rating Sum of Boxes.

The SBF model showed modest out‐of‐sample performance, with predicted and observed CDR‐SOB correlating at *r* = 0.21 in the independent test set (*N* = 41; Figure 2A shows the prediction weight distribution across all 50 components; Figure 2B shows modality weight distributions within each latent component), which falls within the range observed across repeated runs with different random seeds (*r* = 0.21–0.39; mean = 0.31). After false discovery rate multiple comparison, most of the components (90%; 45/50) showed a significant correlation with the target variable (i.e., CDR‐SOB; Figure 2C), which further supports the robustness of our model prediction. The sensitivity analyses across nine dimensionality configurations (10–50 components) and five random initialization rounds confirmed the stable results (see Material SG, Figure S3 in supporting information).

This investigation aimed to apply SBF to investigate the A–T–N framework for AD, specifically for identifying multi‐modal brain patterns that are predictive of clinical diagnosis and *APOE* ε4 carrier status. SBF predicts continuous variables, so CDR‐SOB, which is related to both diagnostic and *APOE* ε4 status, was used as the continuous target variable. Although diagnostic/*APOE* ε4 status were not included in the training stage, SBF is effective for generating a representation space that has high predictive power for variables that are related to the training target ^24^.

### 3.3 SBF latent loadings outperform comparator models for diagnostic prediction

**FIGURE 3:**
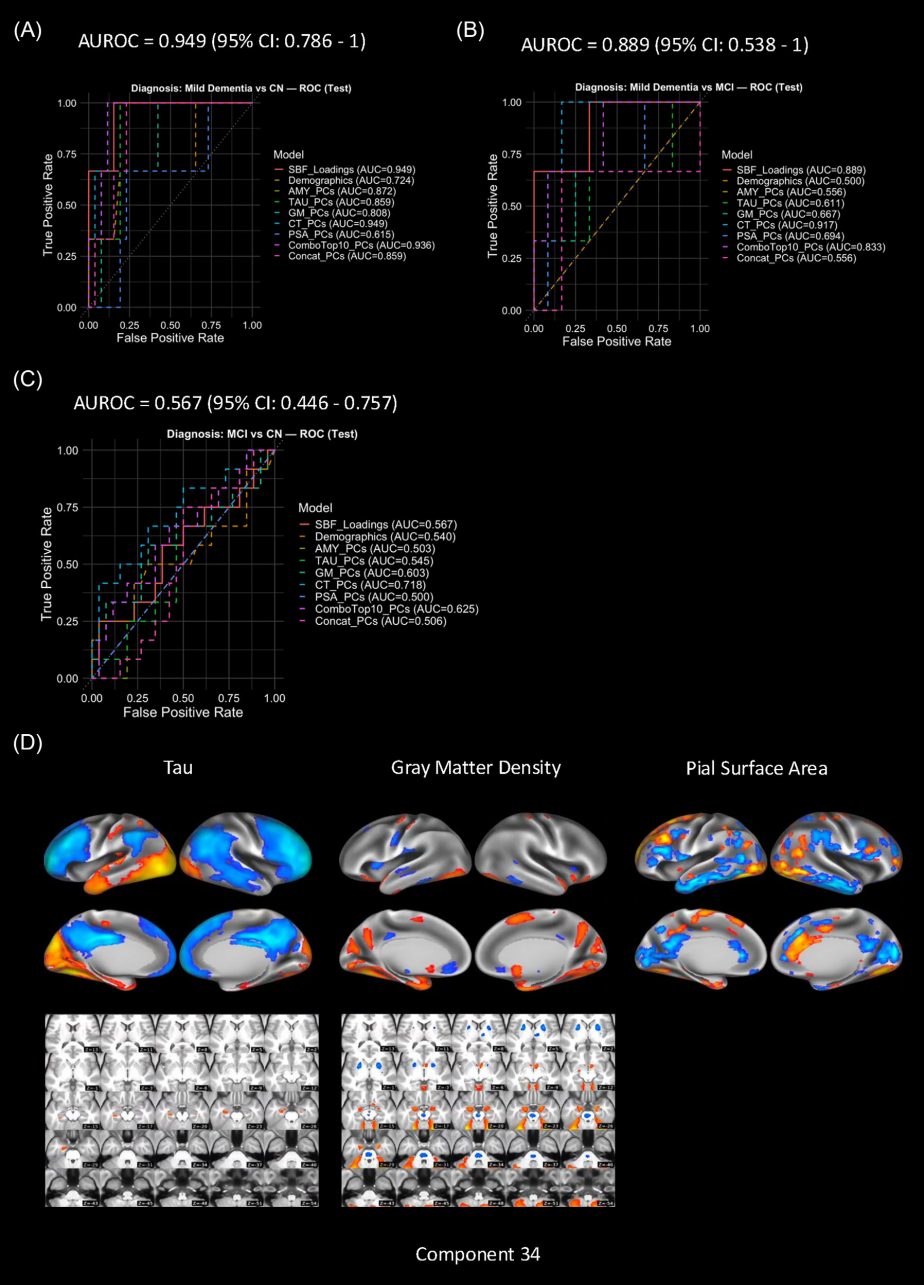
Model performance in predicting clinical diagnoses. A, ROC curve for CN versus mild dementia. B, ROC curve for MCI versus mild dementia. C, ROC curve for CN versus MCI. D, Spatial map of Component 34, the most predictive latent component throughout cognitive decline (for CN vs. MCI, MCI vs. mild dementia, and CN vs. mild dementia), reflecting a multimodal tau–gray matter density–pial surface area pattern. The spatial pattern has negative loadings and was inverted here to make it more intuitive; that is, the pattern strengthens with progression to MCI and mild dementia. Posterior temporal–occipital–dominant tau pattern (right > left) opposing relatively spared posterior default mode and anterior prefrontal regions, with aligned gray matter and surface area changes along the same axis. Upper row (left to right): cortical view for tau, gray matter density, and pial surface area. Lower row (left to right): subcortical view for tau and gray matter density. In (A, B, C), the AUROC listed on top of the legend (red line) corresponds to the SBF loadings–based model. AMY, amyloid positron emission tomography Centiloid; AUC, area under the curve; AUROC, area under the receiver operating characteristic curve; CI, confidence interval; CN, cognitively normal; CT, cortical thickness; GM, gray matter density; MCI, mild cognitive impairment; PC, principal component; PSA, pial surface area; ROC, receiver operating characteristic; SBF, SuperBigFLICA; TAU, tau positron emission tomography standardized uptake value ratio.

After ComBat harmonization, the SBF loadings‐based model showed the strongest overall classification performance across comparator models—including prediction from demographics, single‐modality PCA/ICA, and naïve‐fusion PCA/ICA—achieving the highest macro‐average area under the receiver operating characteristic (ROC) curve (AUROC) 95% confidence interval (CI; 0.80 [0.59, 0.92]), accuracy (0.85 [0.78, 0.91]), balanced accuracy (0.81 [0.73–0.88]), precision (0.7 [0.55–0.84]), and F1 score (0.71 [0.57–0.81]; Table 2A). While comparator models showed uneven sensitivity–specificity trade‐offs, SBF consistently provided balanced gains across metrics (Table 2A, Material SH, Table S12 in supporting information). Pairwise ROC analyses indicated strong performance for dementia discrimination (AUROC = 0.95 [0.79, 1] for CN vs. mild dementia, 0.89 [0.54, 1] for MCI vs. mild dementia), with weak separation of CN versus MCI (AUROC = 0.57 [0.45, 0.76]; Figure 3A–C; Material SH, Figure S4 in supporting information). Area under the precision‐recall curve (AUPRC) exhibited the same relative performance profile (Table 2A; Material SH, Figures S4 and S5, Table S12 in supporting information), confirming that the observed discriminative ranking was not driven by test set imbalance. Note that because the highest CDR‐SOB score among participants corresponds to the range categorized as mild dementia in the proposed translation,^27^ we specify the diagnosis of “dementia” as “mild dementia” for clarity; importantly, this reflects the available ADNI‐3 sample with complete multimodal data rather than exclusion of participants with more advanced dementia.

**TABLE 2:** Model performance of SBF loadings–based models and baselines.

Table 2A. Model performance in predicting clinical diagnoses.								
Model	AUROC (test)	AUPRC (test)	Accuracy (CV)	Balanced accuracy (CV)	Sensitivity (CV)	Specificity (CV)	Precision (CV)	F1 (CV)
SBF loadings	0.80 [0.59, 0.92]	**0.63 [0.12, 0.85]**	**0.85 [0.78, 0.91]**	0.81 [0.73, 0.88]	0.73 [0.57, 0.85]	**0.90 [0.82, 0.95]**	**0.70 [0.55, 0.84]**	**0.71 [0.57, 0.81]**
Demographics	0.59 [0.46, 0.76]	0.38 [0.10, 0.71]	0.57 [0.48, 0.66]	0.63 [0.57, 0.68]	**0.85 [0.75, 0.94]**	0.40 [0.34, 0.46]	0.37 [0.26, 0.48]	0.51 [0.39, 0.63]
Amyloid PCs	0.64 [0.51, 0.91]	0.28 [0.09, 0.80]	0.77 [0.69, 0.84]	0.72 [0.62, 0.81]	0.63 [0.46, 0.78]	0.82 [0.73, 0.90]	0.58 [0.41, 0.73]	0.60 [0.45, 0.72]
Tau PCs	0.67 [0.51, 0.91]	0.37 [0.10, 0.86]	0.78 [0.70, 0.85]	0.73 [0.64, 0.81]	0.56 [0.39, 0.72]	**0.90 [0.83, 0.95]**	0.65 [0.47, 0.81]	0.57 [0.40, 0.71]
Gray matter PCs	0.69 [0.47, 0.94]	0.35 [0.11, 0.91]	0.78 [0.70, 0.85]	0.74 [0.65, 0.82]	0.59 [0.43, 0.74]	0.89 [0.83, 0.93]	0.66 [0.50, 0.79]	0.57 [0.41, 0.70]
Cortical thickness PCs	**0.86 [0.67, 0.96]**	0.54 [0.15, 0.79]	0.81 [0.73, 0.88]	0.74 [0.66, 0.83]	0.62 [0.47, 0.76]	0.87 [0.79, 0.94]	0.61 [0.42, 0.79]	0.59 [0.43, 0.72]
Pial surface area PCs	0.60 [0.45, 0.81]	0.20 [0.08, 0.43]	0.49 [0.40, 0.58]	0.59 [0.52, 0.65]	0.83 [0.73, 0.92]	0.34 [0.26, 0.42]	0.35 [0.24, 0.45]	0.47 [0.35, 0.58]
Top 10 PCs combo	0.80 [0.58, 0.94]	0.56 [0.15, 0.90]	**0.85 [0.78, 0.91]**	**0.82 [0.76, 0.88]**	0.77 [0.67, 0.86]	0.87 [0.80, 0.94]	0.67 [0.51, 0.82]	**0.71 [0.58, 0.82]**
Concatenated PCs	0.64 [0.50, 0.91]	0.28 [0.08, 0.80]	0.77 [0.69, 0.84]	0.72 [0.62, 0.81]	0.61 [0.44, 0.76]	0.83 [0.74, 0.91]	0.59 [0.42, 0.75]	0.59 [0.44, 0.72]

Table 2B. Model performance in predicting *APOE* ε4 status.								
Model	AUROC (test)	AUPRC (test)	Accuracy (CV)	Balanced accuracy (CV)	Sensitivity (CV)	Specificity (CV)	Precision (CV)	F1 (CV)
SBF loadings	**0.82 [0.67, 0.93]**	**0.75 [0.52, 0.94]**	**0.70 [0.64, 0.77]**	**0.67 [0.61, 0.74]**	0.53 [0.42, 0.64]	0.82 [0.75, 0.88]	0.66 [0.54, 0.77]	0.58 [0.49, 0.68]
Demographics	0.47 [0.30, 0.64]	0.42 [0.27, 0.62]	0.61 [0.54, 0.68]	0.52 [0.50, 0.54]	0.04 [0.00, 0.09]	**0.99 [0.97, 1.00]**	**0.75 [0.00, 1.00]**	0.07 [0.00, 0.16]
Amyloid PCs	0.81 [0.66, 0.93]	0.74 [0.51, 0.94]	0.69 [0.63, 0.76]	**0.67 [0.60, 0.74]**	0.55 [0.44, 0.66]	0.79 [0.72, 0.86]	0.64 [0.52, 0.74]	**0.59 [0.49, 0.68]**
Tau PCs	0.71 [0.52, 0.86]	0.74 [0.50, 0.89]	0.61 [0.54, 0.68]	0.53 [0.49, 0.57]	0.12 [0.05, 0.19]	0.94 [0.89, 0.98]	0.56 [0.28, 0.80]	0.20 [0.09, 0.30]
Gray matter PCs	0.53 [0.35, 0.72]	0.44 [0.28, 0.66]	0.44 [0.37, 0.52]	0.53 [0.50, 0.56]	**0.96 [0.91, 1.00]**	0.10 [0.05, 0.15]	0.41 [0.34, 0.48]	0.58 [0.50, 0.65]
Cortical thickness PCs	0.59 [0.41, 0.75]	0.62 [0.39, 0.79]	0.45 [0.38, 0.52]	0.53 [0.50, 0.57]	**0.96 [0.91, 1.00]**	0.11 [0.05, 0.16]	0.42 [0.35, 0.49]	0.58 [0.51, 0.65]
Pial surface area PCs	0.47 [0.29, 0.65]	0.51 [0.27, 0.71]	0.42 [0.35, 0.49]	0.51 [0.48, 0.54]	**0.96 [0.91, 1.00]**	0.06 [0.02, 0.11]	0.41 [0.33, 0.47]	0.57 [0.49, 0.64]
Top 10 PCs combo	0.81 [0.66, 0.93]	0.74 [0.51, 0.94]	0.69 [0.63, 0.76]	**0.67 [0.60, 0.74]**	0.55 [0.44, 0.66]	0.79 [0.72, 0.86]	0.64 [0.52, 0.74]	**0.59 [0.49, 0.68]**
Concatenated PCs	0.81 [0.66, 0.93]	0.74 [0.51, 0.94]	0.69 [0.63, 0.76]	**0.67 [0.60, 0.74]**	0.55 [0.44, 0.66]	0.79 [0.72, 0.86]	0.64 [0.52, 0.74]	**0.59 [0.49, 0.68]**

Notes: In part A, all metrics are reported as macro‐averages across pairwise comparisons (CN vs. MCI, CN vs. mild dementia, MCI vs. mild dementia). Values are shown with 95% confidence intervals on the second line of each cell. The best performance under each metric is bolded. CV means the result was from cross‐validated training data. Values are shown with 95% confidence intervals on the second line of each cell. The best performance under each metric is shown in bold font. CV means the result was from cross‐validated training data.

Abbreviations: APOE, apolipoprotein E; AUPRC, area under the precision‐recall curve; AUROC, area under the receiver operating characteristic curve; CN, cognitively normal; CV, cross‐validated; MCI, mild cognitive impairment; PC, principal component; SBF, SuperBigFLICA.

Across all three pairwise diagnostic comparisons (Material SH, Tables S13–S18 in supporting information), Component 34 emerged as the most discriminative feature—a joint tau–GM–PSA multimodal pattern (Figure 3D) whose subject loadings showed the strongest association with CDR‐SOB among all components (Figure 2C; Material SH, Figure S6 in supporting information). Notably, loadings on this pattern were negatively associated with CDR‐SOB; for interpretability, the pattern is shown inverted (as the signs can be exchanged across both loadings and patterns). In this orientation, greater cognitive decline is characterized by tau accumulation in temporal and occipital cortices (right > left), with relative sparing of posterior default mode regions (posterior cingulate, precuneus, angular gyrus) and anterior prefrontal cortex (middle frontal gyrus, frontal pole). This covaries with preserved GM in the cerebellum, temporal pole, cuneus/lingual gyrus, supplementary motor area, and subgenual cingulate (BA25), alongside atrophy in the brainstem (midbrain, pons), posterior cingulate, and medial frontal cortex. This molecular–structural alteration pattern is further linked to surface area changes, with expansion in middle frontal, precentral, right supramarginal, posterior cingulate, occipital pole, and lingual regions, as well as left lateral occipital and middle temporal cortex, contrasted with contraction in inferior and middle temporal gyri, left supramarginal and angular gyrus, inferior frontal gyrus, frontal pole, and orbitofrontal cortex.

### 3.4 SBF latent loadings outperform comparator models in *APOE* ε4 prediction, driven by an amyloid pattern

**FIGURE 4:**
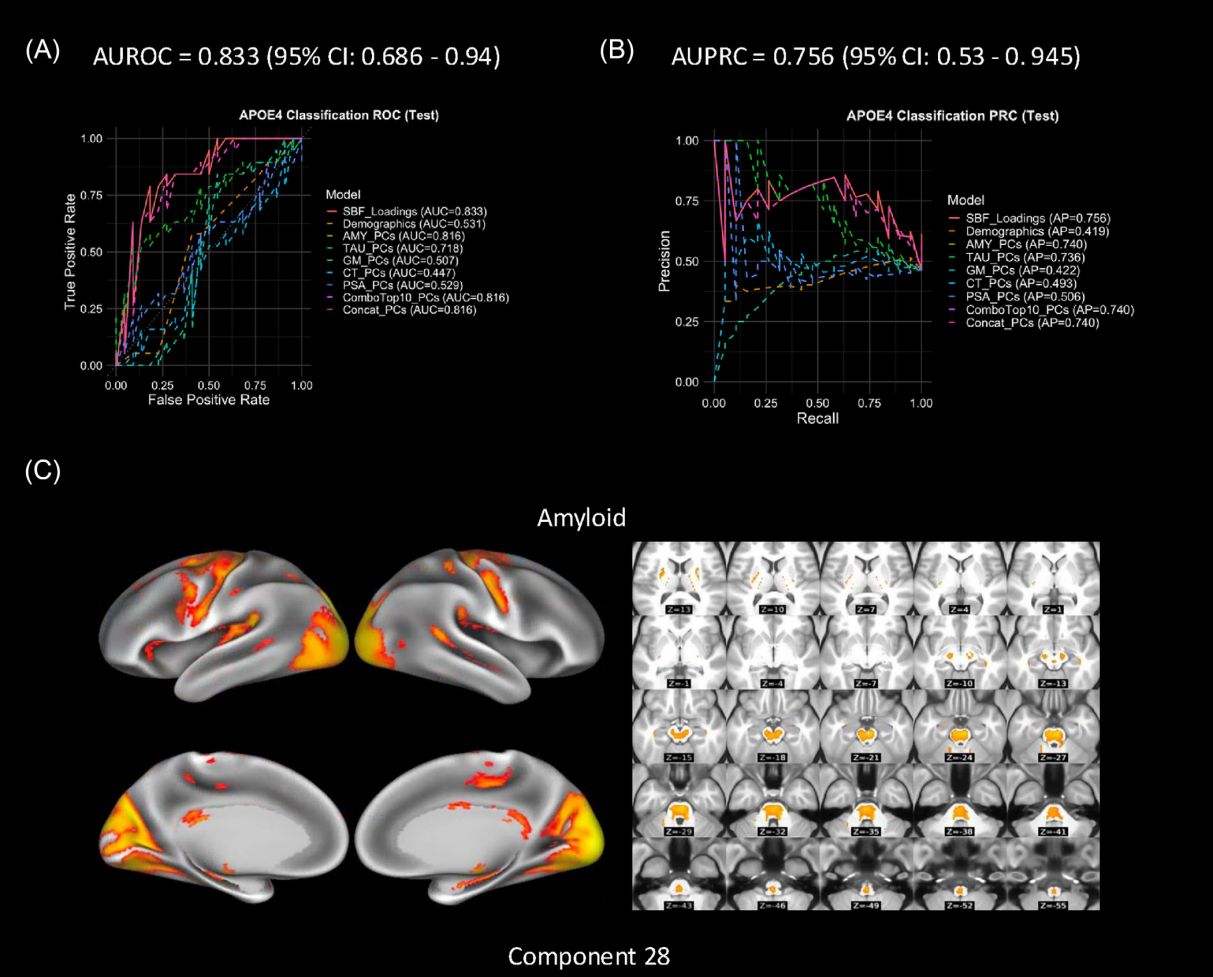
Model performance in predicting *APOE* ε4 status. A, ROC curve for *APOE* ε4 carrier versus non‐carrier. B, PRC curve for *APOE* ε4 carrier versus non‐carrier. C, Spatial map of Component 28, the most predictive component for *APOE* ε4 carrier versus non‐carrier, reflecting the same amyloid‐dominant pattern that best discriminated CN and MCI from early dementia. The pattern was shown inverted for better interpretability. Left, cortical view. Right, subcortical view. In (A, B), the AUROC and AUPRC listed on top of the legend (red line) corresponds to the SBF loadings–based model. AMY, amyloid positron emission tomography Centiloid; *APOE*, apolipoprotein E; AUPRC, area under the precision‐recall curve; AUROC, area under the receiver operating characteristic curve; CI, confidence interval; CN, cognitively normal; GM, gray matter density; MCI, mild cognitive impairment; PC, principal component; PRC, precision‐recall curve; PSA, pial surface area; ROC, receiver operating characteristic; SBF, SuperBigFLICA; TAU, tau positron emission tomography standardized uptake value ratio.

The SBF loadings‐based model showed strong performance in predicting *APOE* ε4 carrier status (AUROC = 0.82 [0.67–0.93]; AUPRC = 0.75 [0.52–0.94]), outperforming demographics and most single‐modality and naïve fusion PCA/ICA comparators (Figure 4A–B; Table 2B; Material SH, Figure S7 and Table S19 in supporting information). Accuracy (0.70 [0.64–0.77]) and balanced accuracy (0.67 [0.61–0.74]) were also among the highest across models, indicating consistently strong discrimination. The most predictive and the only selected SBF feature for *APOE* ε4 carrier status was Component 28, an amyloid‐dominant pattern characterized by prominent involvement of occipital and parietal cortices, with additional contributions from brainstem and cerebellar regions (Figure 4C; the pattern is shown inverted to align with amyloid accumulation across disease stages). Greater expression of this component was associated with *APOE* ε4 carrier status and progression from CN to dementia, consistent with higher occipital amyloid in *APOE* ε4 carriers at earlier stages and a sharper increase from MCI to dementia in non‐carriers. Detailed characterization and validation analyses are provided in Material SI (Figure S8 in supporting information).

### 3.5 Component loadings capture distinct CSF biomarker associations

Among the 192 training participants, 148 (77.1%) had CSF AD biomarkers available for correlation analyses. Loadings for Component 34 were positively correlated with CSF Aβ42 (*r* = 0.28, *p* < .001) and negatively correlated with CSF p‐tau181 (*r* = −0.40, *p* < .001). Interpreted in the orientation in which greater component expression corresponds to greater cognitive decline, this pattern is consistent with increased brain amyloid deposition (lower CSF Aβ42) and higher soluble p‐tau (higher CSF p‐tau181). In contrast, loadings on Component 28 showed the opposite pattern: negative correlation with CSF Aβ42 (*r* = −0.49, *p* < .001) and positive correlation with CSF p‐tau181 (*r* = 0.56, *p* < .001), consistent with greater amyloid pathology and higher p‐tau burden.

## 4 DISCUSSION

We applied SBF, a semi‐supervised multimodal data fusion framework, to investigate the A–T–N framework across the AD continuum. Unlike single‐modality or naïve‐fusion approaches, SBF identifies latent components that simultaneously maximize data reconstruction and non‐imaging phenotype predictions. This enables integration of imaging with cognitive and behavioral measures, enhancing interpretability of multimodal patterns. Using ADNI‐3 data across five imaging modalities (amyloid PET, tau PET, CM density, CT, and PSA), we show SBF captures A–T–N brain–behavior relationships when trained on cognitive decline (CDR‐SOB; out‐of‐sample *r* = 0.21), underscoring disease‐relevant signals. We then used component loadings to estimate cross‐sectional clinical outcomes (i.e., out‐of‐sample prediction, not longitudinal forecasting), including diagnoses and *APOE* ε4 carrier status. In both cases, SBF‐derived models were among the strongest performers, outperforming most single‐modality and unsupervised fusion models (PCA/ICA), indicating capture of disease‐related variation beyond the training target. These findings demonstrate that SBF not only improves prediction of its training target but also uncovers a high‐dimensional representational space that generalizes to related phenotypes. Predicting continuous scores requires finer‐grained information than classification; thus, modest correlations can coexist with strong classification when representations generalize. The out‐of‐sample correlation (*r* = 0.21) is modest, consistent with typical brain–behavior effect sizes and a small test sample.^33^

SBF maintains interpretability because the projection from subject loadings to modality‐specific spatial maps is fully linear, rather than a black‐box model. This interpretability enables explainability: spatial patterns supporting a prediction can be examined directly. As a result, SBF not only predicts a neuropsychological score but also reveals the unimodal or multimodal spatial patterns associated with that target, rather than capturing unrelated intersubject variability, enabling principled inference about dominant neuroimaging signatures driving the outcome. This is an advantage over unguided data‐fusion frameworks, which may capture mixed variance sources and require post hoc isolation of disease‐related effects.

Single‐modality comparators revealed a stage‐dependent pattern aligned with the A–T–N framework; amyloid and tau captured early pathological signals that distinguish clinically distant stages (CN vs. mild dementia; area under the curve [AUC]: amyloid = 0.872, tau = 0.846) but provide poorer resolution between adjacent stages, both earlier (CN vs. MCI; amyloid = 0.526, tau = 0.529) and later (MCI vs. mild dementia; amyloid = 0.556, tau = 0.611). In contrast, CT—a marker of neurodegeneration—more closely tracks clinical progression, consistent with prior findings that regional thinning correlates with symptom severity and is detectable even in asymptomatic amyloid‐positive individuals.^34^ It showed consistently stronger performance, particularly for MCI versus mild dementia (AUC = 0.917) and CN versus mild dementia (AUC = 0.949), with this advantage retained after age residualization (Material SJ, Figure S9 in supporting information). Due to the small independent test set (*N* = 41) and further‐reduced subgroup sizes for some comparisons, formal statistical comparisons of classifier performance (e.g., DeLong test) are not feasible; we therefore reported descriptive metrics only.

Across all pairwise diagnostic classifications, SBF identified Component 34—a tri‐modal tau–neurodegeneration pattern—as the most predictive feature, supporting its relevance as a convergent marker across clinical progression. Within this component, PSA loaded on the same latent disease‐related axis as molecular and volumetric measures, indicating that, despite weaker associations as a standalone feature.^35^ it contributes meaningful disease‐related signal when integrated with tau and GM. At the component level, PSA covaried with tau, but showed a distinct pattern: PSA showed areal expansion in frontal, posterior cingulate, and occipital regions, alongside contraction in temporal, angular, and orbitofrontal cortices. This subregional differentiation indicates that, while jointly expressed, tau and PSA index partially distinct yet coordinated aspects of brain organization.

Despite Component 34's consistent dominance, CN–MCI separation remained the most challenging classification. Its uniformly weaker performance underscores the difficulty of detecting early cognitive decline. Consistent with this, clinicians reported low confidence in identifying MCI in practice,^36^, ^37^ and variability in neuroimaging biomarkers causes considerable overlap with healthy individuals, limiting reliable discrimination between CN and MCI.^38^

Beyond diagnostic classification, distinct components mapped onto biological and genetic axes of disease. Component 34 loadings were associated with CSF amyloid and tau and tracked cognitive decline, reinforcing its interpretation as a progression‐sensitive marker. In contrast, Component 28—an amyloid‐dominant pattern—emerged as the strongest feature for *APOE* ε4 classification, consistent with strong amyloid ICA performance, suggesting that *APOE*‐related variation is primarily captured by amyloid, in line with prior work.^39^ Component 28 also exhibited stage‐dependent differences by *APOE* ε4 status, with stronger expression in non‐carriers at later stages (MCI to mild dementia). This pattern suggests increasing posterior amyloid signals with disease progression, aligning with evidence that spatial distributions of amyloid^40^ and tau^41^ provide critical information beyond global burden for characterizing progression.

We emphasize that SBF is intended primarily as a multimodal research framework for mechanistic discovery, not a clinical diagnostic tool. While the field increasingly seeks simple single‐modality biomarkers, AD is biologically heterogeneous, and a single marker is unlikely to capture the full spectrum of relevant pathophysiology^42^. Importantly, multimodal imaging is often already available in clinical AD evaluation: patients undergoing amyloid PET typically also have structural T1‐weighted MRI, enabling derivation of complementary structural measures. By integrating PET and T1‐derived features, SBF identifies latent patterns reflecting converging disease mechanisms and yields more robust signatures than single modalities. The framework also accommodates missing modalities within a subject, enabling application to more clinically realistic datasets. Thus, SBF is designed to complement—not replace—single‐biomarker strategies.

Looking ahead, SBF's interpretability makes it well suited for expanding to additional modalities (e.g., functional and structural connectivity) to enhance predictive power and mechanistic insight. Key limitations include the cross‐sectional design; future longitudinal applications could clarify temporal disease dynamics. A second limitation is the modest sample size for prediction. However, SBF exploits joint information among modalities within participants and embeds prediction directly into the decomposition to identify multimodal maps that prioritize explaining variance in the target variable. Combined with rigorous methods for cross‐validation and inclusion of a small independent test sample greatly improves the rigor of our findings. Third, although SBF subject loadings carried some detectable scanner‐manufacturer effects, ComBat harmonization reduced this to near chance while leaving classification performance unchanged (Material SC.3 Tables S4–S11), suggesting that SBF's clinical associations reflect genuine biology rather than scanner confounds; future studies with larger, scanner‐diverse cohorts should consider incorporating scanner covariates during model training.

## 5 CONCLUSIONS

Applying SBF to investigate the A–T–N framework for AD demonstrates that semi‐supervised multimodal fusion can capture continuous individual variation (e.g., cognitive decline), while also uncovering integrative brain patterns linked to diagnosis and genetic risk. This highlights SBF's methodological value for heterogeneous, multimodal disease settings, in which interpretability is integral rather than sacrificed for predictive performance. Moving forward, such approaches hold promise for advancing precision staging and guiding targeted interventions in dementia research and care.

## CONFLICT OF INTEREST STATEMENT

The authors declare no competing financial interests or personal relationships that could have influenced the work reported in this study. Author disclosures are available in the Supporting Information.

## CONSENT STATEMENT

All participants provided written informed consent, and each ADNI site obtained local institutional review board approval for study procedures.

## CODE AVAILABILITY

All code for multimodal data fusion and model training, along with the corresponding model cards, is publicly available on GitHub https://github.com/ANSR‐laboratory/SuperBigFLICA_McL.

## Supporting information



Supporting Information

Supporting Information

## Data Availability

Neuroimaging and clinical data were obtained from the Alzheimer's Disease Neuroimaging Initiative (ADNI; http://adni.loni.usc.edu) and can be accessed upon approval of a data use application.
