# Supplementary material for: Investigating the amyloid–tau–neurodegeneration framework in Alzheimer's disease using semi‐supervised multimodal imaging data fusion
Source: Alzheimers Dement (Amst). 2026 May 21;18(2):e70360. doi: 10.1002/dad2.70360 (PMC13239803; doi:10.1002/dad2.70360)
Supplement: Supplementary file 1 — Supporting Information [file DAD2-18-e70360-s002.docx]

**Supplementary Material**

**Table of Contents**

[A. Study Methods 3](#_Toc226105382)

[1 Study participants 3](#_Toc226105383)

[2 Image acquisition and preprocessing 3](#_Toc226105384)

[3 fMRIPrep Methods 6](#_Toc226105385)

[Anatomical data preprocessing 6](#_Toc226105386)

[B. SuperBigFLICA model specification 9](#_Toc226105387)

[C. Scanner Manufacturer Harmonization 10](#_Toc226105388)

[1 Scanner Distribution 10](#_Toc226105389)

[Table S1. MRI scanner manufacturer distribution by split 10](#_Toc226105390)

[Table S2. PET scanner manufacturer distribution by split 11](#_Toc226105391)

[2 ComBat Harmonization Procedure 11](#_Toc226105392)

[3 Scanner Decodability 11](#_Toc226105393)

[Table S3. MRI scanner decodability before/after ComBat 12](#_Toc226105394)

[Table S4. MRI scanner decodability by representation and modality 12](#_Toc226105395)

[Figure S1. Per-component variance explained by scanner manufacturer 13](#_Toc226105396)

[4 Preservation of Clinical Signal 14](#_Toc226105397)

[Table S5. CDR-SOB association preservation before/after ComBat 14](#_Toc226105398)

[5 Pre-Harmonization Classification Results 14](#_Toc226105399)

[Table S6. Diagnosis classification: PCA, pre- vs. post-ComBat 14](#_Toc226105400)

[Table S7. Diagnosis classification: ICA, pre- vs. post-ComBat 15](#_Toc226105401)

[Table S8. CN vs. MCI: PCA, pre- vs. post-ComBat 15](#_Toc226105402)

[Table S9. CN vs. MCI: ICA, pre- vs. post-ComBat 16](#_Toc226105403)

[Table S10. APOE4 classification: PCA, pre- vs. post-ComBat 17](#_Toc226105404)

[Table S11. APOE4 classification: ICA, pre- vs. post-ComBat 17](#_Toc226105405)

[6 Component 7 18](#_Toc226105406)

[Figure S2. Spatial map of Component 7 19](#_Toc226105407)

[D. Classification Models of Clinical Outcomes 20](#_Toc226105408)

[E. Comparator Models 20](#_Toc226105409)

[F. Study participants 21](#_Toc226105410)

[G. Sensitivity of CDR-SOB Prediction to Dimensionality and Random Initialization 21](#_Toc226105411)

[Figure S3. CDR-SOB prediction by dimensionality and random initialization 22](#_Toc226105412)

[H. Classification Tables and Figures 23](#_Toc226105413)

[Figure S4. Diagnosis prediction: SBF, demographics, and ICA models 23](#_Toc226105414)

[Figure S5. Diagnosis prediction: PCA-based feature loadings 24](#_Toc226105415)

[Figure S6. Feature importance for diagnosis classification 25](#_Toc226105416)

[Figure S7. APOE4 prediction: SBF, demographics, and ICA models 26](#_Toc226105417)

[Table S12. Diagnosis classification: SBF vs. ICA comparators (macro-averaged) 27](#_Toc226105418)

[Table S13. CN vs. MCI: SBF vs. ICA comparators 28](#_Toc226105419)

[Table S14. CN vs. Mild Dementia: SBF vs. ICA comparators 29](#_Toc226105420)

[Table S15. MCI vs. Mild Dementia: SBF vs. ICA comparators 30](#_Toc226105421)

[Table S16. CN vs. MCI: SBF vs. PCA comparators 31](#_Toc226105422)

[Table S17. CN vs. Mild Dementia: SBF vs. PCA comparators 32](#_Toc226105423)

[Table S18. MCI vs. Mild Dementia: SBF vs. PCA comparators 33](#_Toc226105424)

[Table S19. APOE4 classification: SBF vs. ICA comparators 33](#_Toc226105425)

[I. Component 28 Validation: Amyloid Quantification and *APOE4* Carrier Status 35](#_Toc226105426)

[Figure S8. Component Centiloid measures by diagnosis and APOE4 status 35](#_Toc226105427)

[J. Age Confound Sensitivity Analysis 36](#_Toc226105428)

[Figure S9. Age residualization sensitivity analysis 38](#_Toc226105429)

# A. Study Methods

## 1 Study participants

Data used in the preparation of this article were obtained from the Alzheimer’s Disease Neuroimaging Initiative (ADNI) database (adni.loni.usc.edu). The ADNI was launched in 2003 as a public-private partnership, led by Principal Investigator Michael W. Weiner, MD. The primary goal of ADNI has been to test whether longitudinal MRI, PET, other biological markers, and clinical and neuropsychological assessment can be used to track the progression of MCI and early AD.

In addition to the eligibility criteria mentioned in the All ADNI‑3 enrollees satisfy a common set of eligibility rules (Alzheimer’s Disease Neuroimaging Initiative, 2016). Briefly, participants are 55–90 years old, have a Geriatric Depression Scale (GDS) score < 6, at least a sixth‑grade education (or equivalent work history), and adequate vision and hearing for neuropsychological testing. Exclusion criteria include major neurologic disorders other than suspected AD (e.g., Parkinson’s disease, hydrocephalus, or significant head trauma), MRI contraindications, recent major psychiatric illness or substance abuse, clinically significant systemic disease, residence in a skilled‑nursing facility, and use of prohibited psychoactive or anticoagulant medications. Cerebrospinal fluid (CSF) samples, when available, were included if collected within one year of the scan were assessed with Roche’s Elecsys assays.

## 2 Image acquisition and preprocessing

In ADNI-3, imaging followed standardized protocols to ensure consistency across sites (Alzheimer’s Disease Neuroimaging Initiative, 2016b). Structural MRI used 3T scanners with a 3D T1-weighted MPRAGE sequence (Weiner et al., 2016). Amyloid PET employed [^18^F]florbetapir (FBP) or [^18^F]florbetaben (FBB), with acquisitions starting 50 minutes post-injection for FBP and 90 minutes for FBB (four 5-minute frames). Tau PET used [^18^F]AV-1451 (flortaucipir [FTP]), acquired in six 5-minute frames starting 75 minutes post-injection(Alzheimer’s Disease Neuroimaging Initiative, 2016a).

Structural image data preprocessing was performed using fMRIPrep 20.2.7(Esteban, Blair, et al., 2018; Esteban, Markiewicz, et al., 2018), which is based on Nipype 1.7.0 (K. Gorgolewski et al., 2011; K. J. Gorgolewski et al., 2018) (see section 3 below for details). Pre-processed T1-weighted images were used to compute probabilistic gray‑matter maps (GM) using FSL-VBM (Andersson et al., 2007; Douaud et al., 2007; Good et al., 2001; Smith et al., 2004), masked to retain only gray-matter regions, as well as cortical surface maps of cortical thickness (CT) and pial surface area (PSA) using FreeSurfer(Fischl, 2012) for each participant.

For all PET image data, we used preprocessed single-frame Standardized Uptake Value Ratio (SUVR) images downloaded from the LONI repository (https://ida.loni.usc.edu), which were already motion-corrected, frame-averaged, co-registered to each participant’s T1-weighted MRI, intensity-normalized to a cerebellar reference region, and nonlinearly warped to MNI152 space with 2 mm isotropic resolution (Landau et al., 2011, 2016; Landau & Jagust, 2015). To harmonize FBP and FBB SUVR maps, we converted voxel-wise SUVR values to Centiloid units using ADNI-provided transformation equations (Kolibash et al., n.d.). Amyloid PET data were further masked to gray-matter voxels to minimize nonspecific white-matter signal, while tau PET data were masked to gray matter with exclusion of white matter and cerebellum.

**References:**

Alzheimer’s Disease Neuroimaging Initiative. (2016a). *ADNI3 PET Technical Procedures Manual Version 2.0*. https://adni.loni.usc.edu/wp-content/uploads/2012/10/ADNI3_PET-Tech-Manual_V2.0_20161206.pdf

Alzheimer’s Disease Neuroimaging Initiative. (2016b). *Alzheimer’s Disease Neuroimaging Initiative 3 (ADNI3) Protocol*. https://adni.loni.usc.edu/wp-content/themes/freshnews-dev-v2/documents/clinical/ADNI3_Protocol.pdf

Andersson, J. L. R., Jenkinson, M., & Smith, S. (2007). *Non-linear registration aka Spatial normalisation FMRIB Technial Report TR07JA2*.

Douaud, G., Smith, S., Jenkinson, M., Behrens, T., Johansen-Berg, H., Vickers, J., James, S., Voets, N., Watkins, K., Matthews, P. M., & James, A. (2007). Anatomically related grey and white matter abnormalities in adolescent-onset schizophrenia. *Brain : A Journal of Neurology*, *130*(Pt 9), 2375–2386. https://doi.org/10.1093/BRAIN/AWM184

Esteban, O., Blair, R., Markiewicz, C. J., Berleant, S. L., Moodie, C., Ma, F., Isik, A. I., Erramuzpe, A., Kent, J. D., Goncalves, M., DuPre, E., Sitek, K. R., Gomez, D. E. P., Lurie, D. J., Ye, Z., Poldrack, R. A., & Gorgolewski, K. J. (2018). fMRIPrep. *Software*. https://doi.org/10.5281/zenodo.852659

Esteban, O., Markiewicz, C. J., Blair, R. W., Moodie, C. A., Isik, A. I., Erramuzpe, A., Kent, J. D., Goncalves, M., DuPre, E., Snyder, M., Oya, H., Ghosh, S. S., Wright, J., Durnez, J., Poldrack, R. A., & Gorgolewski, K. J. (2018). fMRIPrep: a robust preprocessing pipeline for functional MRI. *Nature Methods 2018 16:1*, *16*(1), 111–116. https://doi.org/10.1038/s41592-018-0235-4

Fischl, B. (2012). FreeSurfer. In *NeuroImage* (Vol. 62, Number 2). https://doi.org/10.1016/j.neuroimage.2012.01.021

Good, C. D., Johnsrude, I. S., Ashburner, J., Henson, R. N. A., Friston, K. J., & Frackowiak, R. S. J. (2001). A voxel-based morphometric study of ageing in 465 normal adult human brains. *NeuroImage*, *14*(1 I), 21–36. https://doi.org/10.1006/nimg.2001.0786

Gorgolewski, K., Burns, C. D., Madison, C., Clark, D., Halchenko, Y. O., Waskom, M. L., & Ghosh, S. (2011). Nipype: a flexible, lightweight and extensible neuroimaging data processing framework in Python. *Frontiers in Neuroinformatics*, *5*, 13. https://doi.org/10.3389/fninf.2011.00013

Gorgolewski, K. J., Esteban, O., Markiewicz, C. J., Ziegler, E., Ellis, D. G., Notter, M. P., Jarecka, D., Johnson, H., Burns, C., Manhães-Savio, A., Hamalainen, C., Yvernault, B., Salo, T., Jordan, K., Goncalves, M., Waskom, M., Clark, D., Wong, J., Loney, F., … Ghosh, S. (2018). Nipype. *Software*. https://doi.org/10.5281/zenodo.596855

Kolibash, S. A., Minhas, D., & Lopresti, B. J. (n.d.). *Centiloid Level-2 Analysis of [18F] Florbetaben (FBB) and [18F] Florbetapir (FBP) PET Image Data using the ADNI Pipeline. 2024*. LONI. Retrieved November 20, 2025, from https://adni.loni.usc.edu/wp-content/themes/freshnews-dev-v2/documents/pet/ADNI%20Centiloids%20Final.pdf

Landau, S., & Jagust, W. (2015). Florbetapir processing methods. *Alzheimer’s Disease Neuroimaging Initiative*.

Landau, S., Koeppe, R., & Jagust, W. (2011). Florbetaben processing and positivity threshold derivation. *Alzheimer’s Dis Neuro-Imaging Initiat*.

Landau, S., Ward, T. J., Murphy, A., & Jagust, W. (2016). Flortaucipir (AV-1451) processing methods. *Alzheimer’s Disease Neuroimaging Initiative*.

Smith, S. M., Jenkinson, M., Woolrich, M. W., Beckmann, C. F., Behrens, T. E. J., Johansen-Berg, H., Bannister, P. R., De Luca, M., Drobnjak, I., Flitney, D. E., Niazy, R. K., Saunders, J., Vickers, J., Zhang, Y., De Stefano, N., Brady, J. M., & Matthews, P. M. (2004). Advances in functional and structural MR image analysis and implementation as FSL. *NeuroImage*, *23*(SUPPL. 1), S208–S219. https://doi.org/10.1016/J.NEUROIMAGE.2004.07.051

Weiner, M. W., Veitch, D. P., Aisen, P. S., Beckett, L. A., Cairns, N. J., Green, R. C., Harvey, D., Jack, C. R., Jagust, W., Morris, J. C., Petersen, R. C., Salazar, J., Saykin, A. J., Shaw, L. M., Toga, A. W., & Trojanowski, J. Q. (2016). The Alzheimer’s Disease Neuroimaging Initiative 3: continued innovation for clinical trial improvement. *Alzheimer’s & Dementia : The Journal of the Alzheimer’s Association*, *13*(5), 561. https://doi.org/10.1016/J.JALZ.2016.10.006

## 3 fMRIPrep Methods

Results included in this manuscript come from preprocessing performed using *fMRIPrep* 20.2.7 (Esteban, Markiewicz, et al. (2018); Esteban, Blair, et al. (2018); RRID:SCR_016216), which is based on *Nipype* 1.7.0 (Gorgolewski et al. (2011); Gorgolewski et al. (2018); RRID:SCR_002502).

Anatomical data preprocessing

A total of 1 T1-weighted (T1w) images were found within the input BIDS dataset.The T1-weighted (T1w) image was corrected for intensity non-uniformity (INU) with N4BiasFieldCorrection (Tustison et al. 2010), distributed with ANTs 2.3.3 (Avants et al. 2008, RRID:SCR_004757), and used as T1w-reference throughout the workflow. The T1w-reference was then skull-stripped with a *Nipype* implementation of the antsBrainExtraction.sh workflow (from ANTs), using OASIS30ANTs as target template. Brain tissue segmentation of cerebrospinal fluid (CSF), white-matter (WM) and gray-matter (GM) was performed on the brain-extracted T1w using fast (FSL 5.0.9, RRID:SCR_002823, Zhang, Brady, and Smith 2001). Brain surfaces were reconstructed using recon-all (FreeSurfer 6.0.1, RRID:SCR_001847, Dale, Fischl, and Sereno 1999), and the brain mask estimated previously was refined with a custom variation of the method to reconcile ANTs-derived and FreeSurfer-derived segmentations of the cortical gray-matter of Mindboggle (RRID:SCR_002438, Klein et al. 2017). Volume-based spatial normalization to two standard spaces (MNI152NLin6Asym, MNI152NLin2009cAsym) was performed through nonlinear registration with antsRegistration (ANTs 2.3.3), using brain-extracted versions of both T1w reference and the T1w template. The following templates were selected for spatial normalization: *FSL’s MNI ICBM 152 non-linear 6th Generation Asymmetric Average Brain Stereotaxic Registration Model* [Evans et al. (2012), RRID:SCR_002823; TemplateFlow ID: MNI152NLin6Asym], *ICBM 152 Nonlinear Asymmetrical template version 2009c* [Fonov et al. (2009), RRID:SCR_008796; TemplateFlow ID: MNI152NLin2009cAsym],

Copyright Waiver

The above boilerplate text was automatically generated by fMRIPrep with the express intention that users should copy and paste this text into their manuscripts *unchanged*. It is released under the [CC0](https://creativecommons.org/publicdomain/zero/1.0/) license.

References

Abraham, Alexandre, Fabian Pedregosa, Michael Eickenberg, Philippe Gervais, Andreas Mueller, Jean Kossaifi, Alexandre Gramfort, Bertrand Thirion, and Gael Varoquaux. 2014. “Machine Learning for Neuroimaging with Scikit-Learn.” *Frontiers in Neuroinformatics* 8. <https://doi.org/10.3389/fninf.2014.00014>.

Avants, B.B., C.L. Epstein, M. Grossman, and J.C. Gee. 2008. “Symmetric Diffeomorphic Image Registration with Cross-Correlation: Evaluating Automated Labeling of Elderly and Neurodegenerative Brain.” *Medical Image Analysis* 12 (1): 26–41. <https://doi.org/10.1016/j.media.2007.06.004>.

Behzadi, Yashar, Khaled Restom, Joy Liau, and Thomas T. Liu. 2007. “A Component Based Noise Correction Method (CompCor) for BOLD and Perfusion Based fMRI.” *NeuroImage* 37 (1): 90–101. <https://doi.org/10.1016/j.neuroimage.2007.04.042>.

Dale, Anders M., Bruce Fischl, and Martin I. Sereno. 1999. “Cortical Surface-Based Analysis: I. Segmentation and Surface Reconstruction.” *NeuroImage* 9 (2): 179–94. <https://doi.org/10.1006/nimg.1998.0395>.

Esteban, Oscar, Ross Blair, Christopher J. Markiewicz, Shoshana L. Berleant, Craig Moodie, Feilong Ma, Ayse Ilkay Isik, et al. 2018. “FMRIPrep.” *Software*. Zenodo. <https://doi.org/10.5281/zenodo.852659>.

Esteban, Oscar, Christopher Markiewicz, Ross W Blair, Craig Moodie, Ayse Ilkay Isik, Asier Erramuzpe Aliaga, James Kent, et al. 2018. “fMRIPrep: A Robust Preprocessing Pipeline for Functional MRI.” *Nature Methods*. <https://doi.org/10.1038/s41592-018-0235-4>.

Evans, AC, AL Janke, DL Collins, and S Baillet. 2012. “Brain Templates and Atlases.” *NeuroImage* 62 (2): 911–22. <https://doi.org/10.1016/j.neuroimage.2012.01.024>.

Fonov, VS, AC Evans, RC McKinstry, CR Almli, and DL Collins. 2009. “Unbiased Nonlinear Average Age-Appropriate Brain Templates from Birth to Adulthood.” *NeuroImage* 47, Supplement 1: S102. <https://doi.org/10.1016/S1053-8119(09)70884-5>.

Gorgolewski, K., C. D. Burns, C. Madison, D. Clark, Y. O. Halchenko, M. L. Waskom, and S. Ghosh. 2011. “Nipype: A Flexible, Lightweight and Extensible Neuroimaging Data Processing Framework in Python.” *Frontiers in Neuroinformatics* 5: 13. <https://doi.org/10.3389/fninf.2011.00013>.

Gorgolewski, Krzysztof J., Oscar Esteban, Christopher J. Markiewicz, Erik Ziegler, David Gage Ellis, Michael Philipp Notter, Dorota Jarecka, et al. 2018. “Nipype.” *Software*. Zenodo. <https://doi.org/10.5281/zenodo.596855>.

Greve, Douglas N, and Bruce Fischl. 2009. “Accurate and Robust Brain Image Alignment Using Boundary-Based Registration.” *NeuroImage* 48 (1): 63–72. <https://doi.org/10.1016/j.neuroimage.2009.06.060>.

Huntenburg, Julia M. 2014. “Evaluating Nonlinear Coregistration of BOLD EPI and T1w Images.” Master’s Thesis, Berlin: Freie Universität. <http://hdl.handle.net/11858/00-001M-0000-002B-1CB5-A>.

Jenkinson, Mark, Peter Bannister, Michael Brady, and Stephen Smith. 2002. “Improved Optimization for the Robust and Accurate Linear Registration and Motion Correction of Brain Images.” *NeuroImage* 17 (2): 825–41. <https://doi.org/10.1006/nimg.2002.1132>.

Klein, Arno, Satrajit S. Ghosh, Forrest S. Bao, Joachim Giard, Yrjö Häme, Eliezer Stavsky, Noah Lee, et al. 2017. “Mindboggling Morphometry of Human Brains.” *PLOS Computational Biology* 13 (2): e1005350. <https://doi.org/10.1371/journal.pcbi.1005350>.

Lanczos, C. 1964. “Evaluation of Noisy Data.” *Journal of the Society for Industrial and Applied Mathematics Series B Numerical Analysis* 1 (1): 76–85. <https://doi.org/10.1137/0701007>.

Power, Jonathan D., Anish Mitra, Timothy O. Laumann, Abraham Z. Snyder, Bradley L. Schlaggar, and Steven E. Petersen. 2014. “Methods to Detect, Characterize, and Remove Motion Artifact in Resting State fMRI.” *NeuroImage* 84 (Supplement C): 320–41. <https://doi.org/10.1016/j.neuroimage.2013.08.048>.

Pruim, Raimon H. R., Maarten Mennes, Daan van Rooij, Alberto Llera, Jan K. Buitelaar, and Christian F. Beckmann. 2015. “ICA-AROMA: A Robust ICA-Based Strategy for Removing Motion Artifacts from fMRI Data.” *NeuroImage* 112 (Supplement C): 267–77. <https://doi.org/10.1016/j.neuroimage.2015.02.064>.

Satterthwaite, Theodore D., Mark A. Elliott, Raphael T. Gerraty, Kosha Ruparel, James Loughead, Monica E. Calkins, Simon B. Eickhoff, et al. 2013. “An improved framework for confound regression and filtering for control of motion artifact in the preprocessing of resting-state functional connectivity data.” *NeuroImage* 64 (1): 240–56. <https://doi.org/10.1016/j.neuroimage.2012.08.052>.

Treiber, Jeffrey Mark, Nathan S. White, Tyler Christian Steed, Hauke Bartsch, Dominic Holland, Nikdokht Farid, Carrie R. McDonald, Bob S. Carter, Anders Martin Dale, and Clark C. Chen. 2016. “Characterization and Correction of Geometric Distortions in 814 Diffusion Weighted Images.” *PLOS ONE* 11 (3): e0152472. <https://doi.org/10.1371/journal.pone.0152472>.

Tustison, N. J., B. B. Avants, P. A. Cook, Y. Zheng, A. Egan, P. A. Yushkevich, and J. C. Gee. 2010. “N4ITK: Improved N3 Bias Correction.” *IEEE Transactions on Medical Imaging* 29 (6): 1310–20. <https://doi.org/10.1109/TMI.2010.2046908>.

Wang, Sijia, Daniel J. Peterson, J. C. Gatenby, Wenbin Li, Thomas J. Grabowski, and Tara M. Madhyastha. 2017. “Evaluation of Field Map and Nonlinear Registration Methods for Correction of Susceptibility Artifacts in Diffusion MRI.” *Frontiers in Neuroinformatics* 11. <https://doi.org/10.3389/fninf.2017.00017>.

Zhang, Y., M. Brady, and S. Smith. 2001. “Segmentation of Brain MR Images Through a Hidden Markov Random Field Model and the Expectation-Maximization Algorithm.” *IEEE Transactions on Medical Imaging* 20 (1): 45–57. <https://doi.org/10.1109/42.906424>.

# B. SuperBigFLICA model specification

SBF models each modality matrix as a product of shared latent factors and modality-specific spatial maps (Gong et al., 2023). For K modalities, the model is:

Eq. 1:

$$X^{\left( k \right)}=ZH^{\left( k \right)}W^{\left( k \right)}+E^{k}, k=1,\ldots,K$$

where Z is the shared subject loading matrix, W^(k)^ contains the modality-specific spatial loadings, H^(k)^ is a positive diagonal modality weighting matrix, and E^(k)^ represents Gaussian residual noise. Simultaneously, SBF predicts the target variable through a linear model:

Eq. 2:

$$Y=ZB+E$$

where B contains prediction weights mapping the shared latent space to the outcome (Gong et al., 2023). SBF jointly minimizes the following multi-objective loss function:

Eq. 3:

$${}_{W, H, B}^{min}{[ \sum_{k=1}^{k} (\lambda_{1}^{\left( k \right)}\left| \left| X^{\left( k \right)}-Z\left( H^{\left( k \right)}W^{\left( k \right)} \right) \right| \right|_{2}^{2}}+ \lambda_{2}^{\left( k \right)}\left| W^{\left( k \right)} \right| ]+[\sum_{i=1}^{Q} \left( \lambda_{3}^{\left( i \right)}\left| \left| Y_{i}-ZB_{i} \right| \right|_{2}^{2} + \lambda_{4}^{\left( i \right)}\left| B_{i} \right| \right)]$$

where the first summation represents the data reconstruction loss across K modalities (L2 reconstruction error with L1 sparsity on spatial maps), and the second summation represents the prediction loss across Q target variables (here Q=1, corresponding to CDR-SOB; L2 prediction error with L1 regularization on prediction weights; in our implementation, an additional L2 penalty was applied to the prediction weights for numerical stability). The λ parameters control the relative weighting of each term. Rather than tuning these weights via cross-validation, SBF adopts a Bayesian perspective in which the λ parameters correspond to precision hyperparameters under Gaussian/Laplacian priors and are estimated jointly via maximum a posteriori (MAP) estimation (Gong et al., 2023). This allows the model to automatically balance reconstruction and prediction losses across modalities. Model parameters (W, H, B) were optimized using RMSprop (momentum = 0.9), while the distributional loss-balancing parameters were optimized using Adam (Kingma & Ba, 2014), following a mini-batch stochastic gradient descent scheme with cosine learning rate annealing. Dropout regularization (p = 0.2) was applied to the input modalities and subject loadings, and batch normalization was applied to subject loadings during training. Prior to SBF decomposition, dictionary learning was applied to each modality matrix to reduce dimensionality from the voxel/vertex level to 250 dictionary atoms, using an L1-penalized objective optimized over 20 iterations with stochastic gradient descent (SGD).

Importantly, because Z is shared between the reconstruction term (Eq. 1) and the prediction term (Eq. 2), the latent factors are optimized to both explain multimodal imaging variance and maximize prediction of CDR-SOB; thus, the supervision directly biases the extracted components toward outcome-relevant variance rather than purely unsupervised covariance structure.

References:

Gong, W., Bai, S., Zheng, Y. Q., Smith, S. M., & Beckmann, C. F. (2023). Supervised Phenotype Discovery from Multimodal Brain Imaging. *IEEE Transactions on Medical Imaging*, *42*(3), 834–849. <https://doi.org/10.1109/TMI.2022.3218720>

Kingma, D. P., & Ba, J. L. (2014). Adam: A Method for Stochastic Optimization. *3rd International Conference on Learning Representations, ICLR 2015 - Conference Track Proceedings*. https://arxiv.org/pdf/1412.6980

# C. Scanner Manufacturer Harmonization

## 1 Scanner Distribution

MRI and PET scanner manufacturer distributions across training and test splits are shown in Tables S1 and S2.

### Table S1. MRI scanner manufacturer distribution by split

Table S1*. MRI scanner manufacturer distribution by split.*

|  | **Siemens** | **GE** | **Philips** | **Total** |
| --- | --- | --- | --- | --- |
| Train | 131 | 28 | 33 | 192 |
| Test | 34 | 7 | 0* | 41 |

*Philips is absent from the test set. The primary ComBat model was estimated on all three manufacturers in training and applied to the test set (Siemens and GE only). A 2-scanner sensitivity analysis (Siemens + GE only) was also performed.

### Table S2. PET scanner manufacturer distribution by split

Table S2*. PET scanner manufacturer distribution by split (matched subjects only).*

|  | **Siemens** | **GE** | **Philips** | **MiE†** | **Matched total‡** |
| --- | --- | --- | --- | --- | --- |
| Train | 101 | 68 | 19 | 2 | 188 |
| Test | 14 | 14 | 13 | 0 | 41 |

†MiE (n = 2 train, n = 0 test) was excluded from PET scanner analyses due to insufficient sample size (minimum threshold: n = 10 per manufacturer).

‡Two training subjects were excluded from PET analyses due to mismatched amyloid and tau PET scanner manufacturers: one with Siemens amyloid / GE tau), the other with GE amyloid / Philips tau.

## 2 ComBat Harmonization Procedure

ComBat harmonization (Johnson et al., 2007) was applied to SBF subject loadings, as well as to per-modality ICA and PCA loadings (AMY, TAU, GM, CT, PSA; 50 components each), using MRI scanner manufacturer (Siemens / GE / Philips) as the batch variable. CDR-SOB was included as a biological covariate to preserve clinically relevant variance during harmonization. ComBat parameters were estimated on the training set only (n = 192) and applied to the test set (n = 41) without refitting, to prevent data leakage. A custom parametric implementation of ComBat was developed to enable this fit/apply split, since the standard sva::ComBat package estimates parameters on the full dataset simultaneously. This implementation was validated against sva::ComBat on the training set, yielding a maximum absolute difference of 0.055 across all components, confirming equivalent results.

The primary analysis used 3-scanner ComBat (Siemens + GE + Philips). A sensitivity analysis used 2-scanner ComBat (Siemens + GE only); results were consistent across both models (Table S3).

## 3 Scanner Decodability

To quantify scanner batch effects, we trained a LASSO logistic regression classifier (Siemens vs. GE, binary) on pre- and post-ComBat loadings, using BIC-based lambda selection and balanced class weights, trained on the training set and evaluated on the held-out test set. Statistical significance was assessed via a permutation test (1,000 permutations): in each permutation, training-set class labels were randomly shuffled, the LASSO classifier was refitted on the shuffled training data using BIC-based lambda selection, and evaluated on the held-out test set; the empirical p-value was the proportion of permuted test-set AUCs that equalled or exceeded the observed AUC.

### Table S3. MRI scanner decodability before/after ComBat

Table S3*. MRI scanner manufacturer decodability before and after ComBat (SBF loadings).*

| **Condition** | **AUC** | **Balanced accuracy** | **Permutation p** | **Median scanner R²** |
| --- | --- | --- | --- | --- |
| Pre-ComBat | 0.769 | 0.697 | 0.035 | 0.037 |
| Post-ComBat (3-scanner) | 0.603 | 0.592 | 0.080 | 0.007 |
| Post-ComBat (2-scanner) | 0.632 | 0.607 | 0.051 | 0.009 |

ComBat substantially reduced scanner decodability in SBF loadings. Residual decodability after harmonization (AUC = 0.603) was non-significant (permutation p = 0.080), indicating no statistically significant scanner signal remained.

**Scanner decodability across ICA and PCA modalities.** The same analysis was applied to all ICA and PCA loading representations. Pre-ComBat scanner AUC values, broken down by modality, are shown in Table S4. Notably, the PET-derived modalities (AMY, TAU) exhibited substantial MRI scanner decodability (ICA AMY: AUC = 0.983; ICA TAU: AUC = 0.874). This is expected: the ADNI-preprocessed SUVR (tau) and Centiloid (amyloid) maps were generated using co-registration of PET images to each participant’s T1-weighted MRI (Coath et al., 2023), meaning that MRI scanner characteristics are propagated into the PET-derived spatial maps during preprocessing. Applying MRI-based ComBat to amyloid and tau PET PCA/IC loadings therefore targets the dominant source of batch effects in those representations. PSA loadings showed no detectable scanner effect pre-ComBat (AUC = 0.500 for both ICA and PCA). Post-ComBat, BIC selected the null model (zero degrees of freedom) for all ICA and PCA modalities, yielding AUC = 0.500 universally.

### Table S4. MRI scanner decodability by representation and modality

Table S4*. Pre- and post-ComBat (3-scanner) MRI scanner decodability (AUC) by representation and modality.*

| **Modality** | **Type** | **ICA pre-AUC** | **ICA post-AUC** | **PCA pre-AUC** | **PCA post-AUC** |
| --- | --- | --- | --- | --- | --- |
| AMY | PET-derived | 0.983 | 0.500 | 0.958 | 0.500 |
| TAU | PET-derived | 0.874 | 0.500 | 0.891 | 0.500 |
| GM | MRI-derived | 0.996 | 0.500 | 0.979 | 0.500 |
| CT | MRI-derived | 1.000 | 0.500 | 0.979 | 0.500 |
| PSA | MRI-derived | 0.500 | 0.500 | 0.500 | 0.500 |

ComBat was applied using MRI scanner manufacturer as the batch variable. For PET-derived modalities (AMY, TAU), the high pre-ComBat MRI scanner AUC reflects that the ADNI-preprocessed PET maps were generated with co-registration to each participant’s T1 MRI, propagating MRI scanner characteristics into the PET-derived representations.

**PET scanner decodability (SBF loadings).** PET scanner manufacturer decodability was additionally tested on SBF loadings using PET manufacturer (GE / Philips / Siemens) as the outcome variable, excluding MiE and mismatched subjects (188 train, 41 test). A multinomial LASSO classifier (BIC-selected) yielded macro-averaged AUC = 0.50, balanced accuracy = 0.333 (chance level for 3 classes), and permutation p = 0.97, indicating no detectable PET-specific scanner signal in SBF loadings. Figure S1 shows the per-component R² explained by MRI and PET manufacturer across all 50 SBF components. MRI manufacturer explained substantially more variance per component (mean R² = 0.033, max = 0.210 at V41) compared to PET manufacturer (mean R² = 0.012, max = 0.027 at V27), consistent with the PET preprocessing pipeline using MRI-based co-registration, which carries MRI scanner characteristics forward into PET-derived representations.


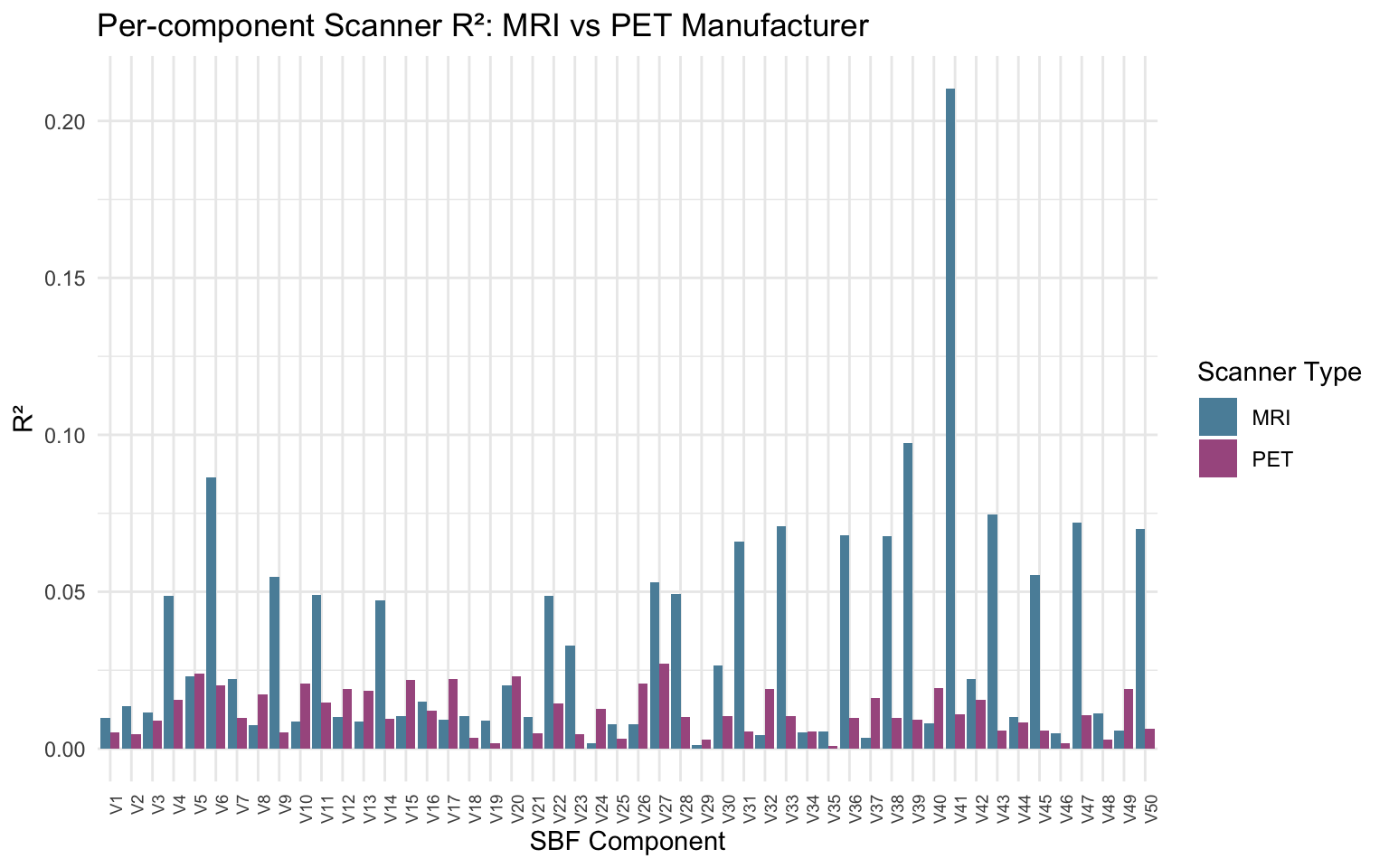


### Figure S1. Per-component variance explained by scanner manufacturer

*Figure S1. Per-component univariate R² explained by MRI manufacturer (blue) and PET manufacturer (purple) for each of the 50 SBF latent components. MRI scanner manufacturer explains substantially more variance (mean R² = 0.033) than PET scanner manufacturer (mean R² = 0.012) across components. V41 shows the largest MRI scanner effect (R² = 0.210); V27 shows the largest PET scanner effect (R² = 0.027).*

## 4 Preservation of Clinical Signal

To verify that ComBat did not remove clinically relevant variance, we compared Spearman correlations between SBF component loadings and CDR-SOB before and after harmonization (training set, FDR-corrected q < 0.05).

### Table S5. CDR-SOB association preservation before/after ComBat

Table S5*. CDR-SOB association preservation before and after ComBat (SBF loadings).*

| **Metric** | **Pre-ComBat** | **Post-ComBat (3-scanner)** |
| --- | --- | --- |
| FDR-significant components (q < 0.05) | 46 / 50 | 45 / 50 |
| Maximum \|r\| with CDR-SOB | 0.614 | 0.614 |

CDR-SOB associations were virtually identical before and after harmonization, confirming that ComBat preserved clinically relevant variance.

## 5 Pre-Harmonization Classification Results

Tables S6–S11 compare diagnosis classification performance before and after ComBat for SBF and single-modality ICA/PCA comparator models (all values are test-set AUC [bootstrap 95% CI], n = 41). Tables S6–S7 show the macro-average one-vs-one AUC; Tables S8–S9 present pairwise comparisons (we report CN vs. MCI here due to more balanced sample sizes, as comparisons involving dementia included only three cases), separately for PCA-based and ICA-based representations. Tables S10–S11 compare *APOE4* carrier classification performance before and after ComBat. SBF pre-ComBat performance was virtually identical to post-ComBat across all comparisons, confirming that ComBat did not remove diagnostically or genetically relevant variance.

### Table S6. Diagnosis classification: PCA, pre- vs. post-ComBat

Table S6*. Diagnosis classification performance (macro-average one-vs-one ROC AUC [bootstrap 95% CI], test set): PCA comparator models, pre- vs post-ComBat (3-scanner).*

| **Model** | **Pre-ComBat AUC [95% CI]** | **Post-ComBat AUC [95% CI]** |
| --- | --- | --- |
| SBF (LASSO) | 0.80 [0.60, 0.92] | 0.80 [0.59, 0.92] |
| CT comparator models | 0.85 [0.69, 0.95] | 0.86 [0.67, 0.96] |
| GM comparator models | 0.72 [0.49, 0.94] | 0.69 [0.47, 0.94] |
| TAU comparator models | 0.67 [0.53, 0.91] | 0.67 [0.51, 0.91] |
| AMY comparator models | 0.66 [0.50, 0.92] | 0.64 [0.51, 0.91] |
| PSA comparator models | 0.60 [0.45, 0.82] | 0.60 [0.45, 0.81] |
| ComboTop10 (naive) | 0.82 [0.62, 0.94] | 0.80 [0.58, 0.94] |
| Concat (naive) | 0.66 [0.50, 0.92] | 0.64 [0.50, 0.91] |

AUC = test-set point estimate [bootstrap 95% CI]. Macro-average across CN vs. MCI, CN vs. Mild Dementia, and MCI vs. Mild Dementia. PCA = 50 principal components per modality. SBF (LASSO) uses the same loadings in both PCA and ICA rows.

### Table S7. Diagnosis classification: ICA, pre- vs. post-ComBat

Table S7*. Diagnosis classification performance (macro-average one-vs-one ROC AUC [bootstrap 95% CI], test set): ICA comparator models, pre- vs post-ComBat (3-scanner).*

| **Model** | **Pre-ComBat AUC [95% CI]** | **Post-ComBat AUC [95% CI]** |
| --- | --- | --- |
| SBF (LASSO) | 0.80 [0.60, 0.92] | 0.80 [0.59, 0.92] |
| CT comparator models | 0.71 [0.60, 0.88] | 0.66 [0.53, 0.90] |
| GM comparator models | 0.70 [0.61, 0.90] | 0.71 [0.62, 0.90] |
| TAU comparator models | 0.62 [0.52, 0.89] | 0.61 [0.44, 0.78] |
| AMY comparator models | 0.66 [0.53, 0.92] | 0.69 [0.55, 0.92] |
| PSA comparator models | 0.69 [0.52, 0.81] | 0.52 [0.49, 0.60] |
| ComboTop10 (naive) | 0.68 [0.47, 0.94] | 0.73 [0.56, 0.94] |
| Concat (naive) | 0.66 [0.56, 0.91] | 0.65 [0.57, 0.74] |

AUC = test-set point estimate [bootstrap 95% CI]. Macro-average across CN vs. MCI, CN vs. Mild Dementia, and MCI vs. Mild Dementia. ICA = 50 independent components per modality. Pre-ComBat ICA bootstrap 95% CIs were recovered by averaging per-comparison CI bounds across the three pairwise comparisons, consistent with the macro-averaging procedure used in all other conditions.

### Table S8. CN vs. MCI: PCA, pre- vs. post-ComBat

Table S8*. CN vs. MCI classification performance (test-set ROC AUC [bootstrap 95% CI]): PCA comparator models, pre- vs post-ComBat.*

| **Model** | **Pre-ComBat AUC [95% CI]** | **Post-ComBat AUC [95% CI]** |
| --- | --- | --- |
| SBF (LASSO) | 0.57 [0.46, 0.77] | 0.57 [0.45, 0.76] |
| CT comparator models | 0.65 [0.46, 0.86] | 0.72 [0.45, 0.88] |
| GM comparator models | 0.62 [0.43, 0.81] | 0.60 [0.47, 0.81] |
| TAU comparator models | 0.62 [0.47, 0.81] | 0.54 [0.40, 0.73] |
| AMY comparator models | 0.57 [0.43, 0.77] | 0.50 [0.44, 0.72] |
| PSA comparator models | 0.50 [0.50, 0.50] | 0.50 [0.50, 0.50] |
| ComboTop10 (naive) | 0.63 [0.45, 0.82] | 0.63 [0.45, 0.81] |
| Concat (naive) | 0.58 [0.43, 0.77] | 0.51 [0.44, 0.72] |

n = 12 MCI (positive class), n = 26 CN (negative class), test set. AUC = test-set point estimate [bootstrap 95% CI].

### Table S9. CN vs. MCI: ICA, pre- vs. post-ComBat

Table S9*. CN vs. MCI classification performance (test-set ROC AUC [bootstrap 95% CI]): ICA comparator models, pre- vs post-ComBat.*

| **Model** | **Pre-ComBat AUC [95% CI]** | **Post-ComBat AUC [95% CI]** |
| --- | --- | --- |
| SBF (LASSO) | 0.57 [0.46, 0.77] | 0.57 [0.45, 0.76] |
| CT comparator models | 0.63 [0.46, 0.83] | 0.66 [0.45, 0.85] |
| GM comparator models | 0.56 [0.42, 0.78] | 0.54 [0.44, 0.78] |
| TAU comparator models | 0.54 [0.44, 0.76] | 0.63 [0.44, 0.83] |
| AMY comparator models | 0.54 [0.44, 0.77] | 0.49 [0.43, 0.76] |
| PSA comparator models | 0.50 [0.50, 0.50] | 0.50 [0.50, 0.50] |
| ComboTop10 (naive) | 0.64 [0.40, 0.82] | 0.66 [0.46, 0.83] |
| Concat (naive) | 0.47 [0.42, 0.74] | 0.53 [0.45, 0.72] |

n = 12 MCI (positive class), n = 26 CN (negative class), test set.

### Table S10. APOE4 classification: PCA, pre- vs. post-ComBat

Table S10*. APOE4 carrier classification performance (test-set ROC AUC [bootstrap 95% CI], n = 41: 19 carriers, 22 non-carriers): PCA comparator models, pre- vs post-ComBat.*

| **Model** | **Pre-ComBat AUC [95% CI]** | **Post-ComBat AUC [95% CI]** |
| --- | --- | --- |
| SBF (LASSO) | 0.83 [0.69, 0.94] | 0.82 [0.67, 0.93] |
| AMY comparator models | 0.82 [0.66, 0.93] | 0.81 [0.66, 0.93] |
| TAU comparator models | 0.72 [0.51, 0.88] | 0.71 [0.52, 0.86] |
| CT comparator models | 0.45 [0.42, 0.72] | 0.59 [0.41, 0.75] |
| GM comparator models | 0.51 [0.45, 0.70] | 0.53 [0.35, 0.72] |
| PSA comparator models | 0.53 [0.44, 0.72] | 0.47 [0.29, 0.65] |
| ComboTop10 (naive) | 0.82 [0.66, 0.93] | 0.81 [0.66, 0.93] |
| Concat (naive) | 0.82 [0.66, 0.93] | 0.81 [0.66, 0.93] |

AUC = test-set point estimate [bootstrap 95% CI]. Positive class: *APOE4* carrier.

### Table S11. APOE4 classification: ICA, pre- vs. post-ComBat

Table S11*. APOE4 carrier classification performance (test-set ROC AUC [bootstrap 95% CI], n = 41: 19 carriers, 22 non-carriers): ICA comparator models, pre- vs post-ComBat.*

| **Model** | **Pre-ComBat AUC [95% CI]** | **Post-ComBat AUC [95% CI]** |
| --- | --- | --- |
| SBF (LASSO) | 0.83 [0.69, 0.94] | 0.82 [0.67, 0.93] |
| AMY comparator models | 0.88 [0.75, 0.97] | 0.89 [0.75, 0.98] |
| TAU comparator models | 0.41 [0.25, 0.63] | 0.57 [0.25, 0.63] |
| CT comparator models | 0.60 [0.42, 0.77] | 0.51 [0.31, 0.66] |
| GM comparator models | 0.51 [0.33, 0.67] | 0.47 [0.30, 0.65] |
| PSA comparator models | 0.54 [0.29, 0.65] | 0.52 [0.31, 0.65] |
| ComboTop10 (naive) | 0.52 [0.32, 0.67] | 0.52 [0.29, 0.68] |
| Concat (naive) | 0.61 [0.41, 0.78] | 0.88 [0.76, 0.97] |

AUC = test-set point estimate [bootstrap 95% CI]. ICA = 50 independent components per modality. SBF uses the same loadings as in Table S10. Positive class: *APOE4* carrier.

## 6 Component 7

Component 7 was the strongest predictor for CN vs. MCI classification in pre-ComBat harmonization analyses. Following ComBat harmonization, Component 7 remains selected as a predictor by the LASSO classifier but is no longer the strongest individual predictor; Component 34 emerges as the most discriminative feature across all three pairwise diagnosis comparisons (see main text Section 3.3).

Within this same latent component, PSA also varied systematically across posterior and association cortices, but emphasized different subregions than tau, with lower surface area in ventral/posterior visual regions (e.g., lingual gyrus and inferior parietal cortex) and relatively greater surface area in lateral occipital and supramarginal areas. This subregional differentiation indicates that, although tau and PSA are jointly expressed at the component level, they index partially distinct but coordinated aspects of brain organization. The pattern showed similar trajectories across *APOE4* groups with no significant interaction (p = 0.247), consistent with stage-sensitive but genotype-independent variation.


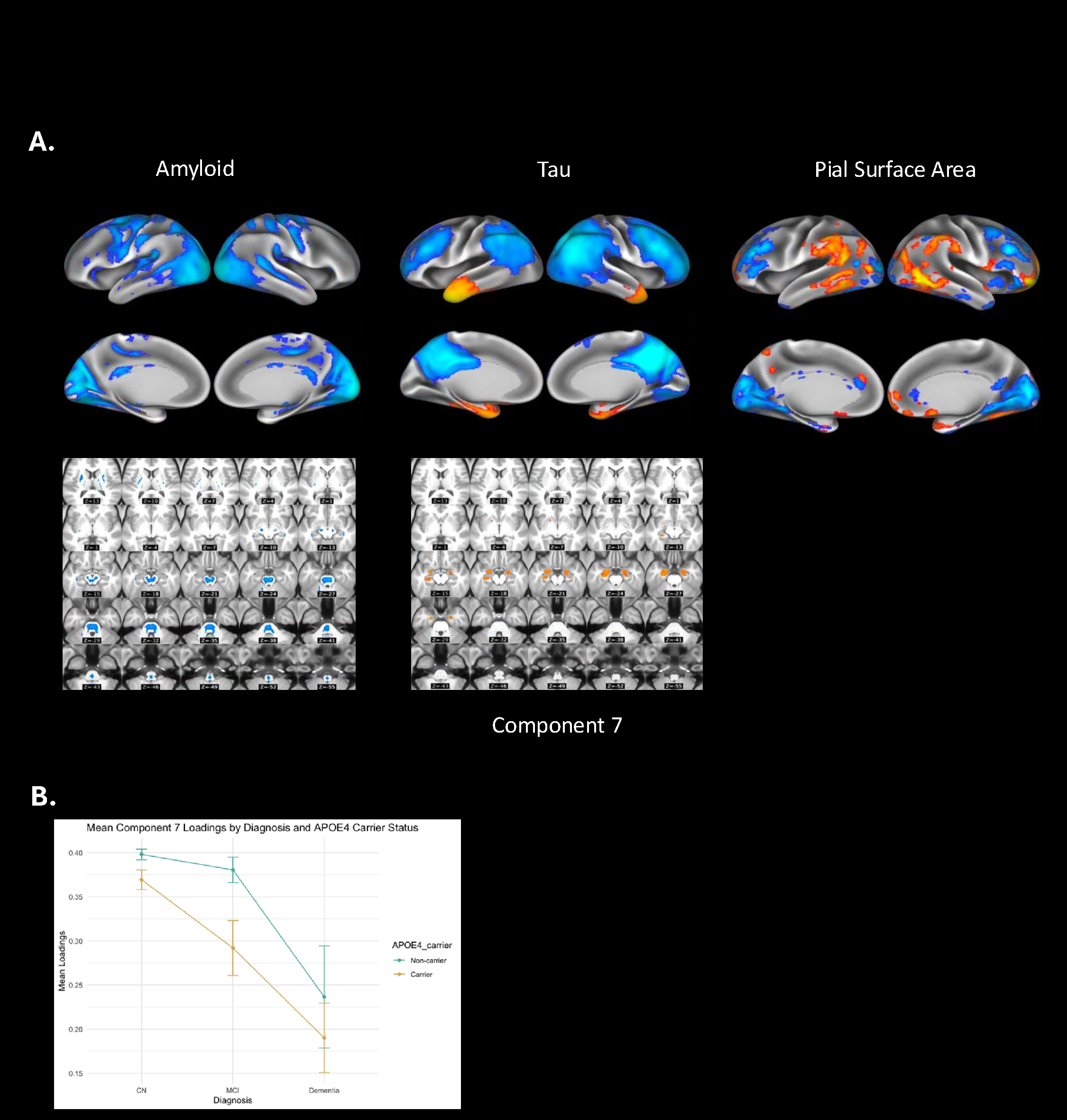


### Figure S2. Spatial map of Component 7

Figure S2. Component 7. (A) Spatial map of Component 7, an A-T-N multimodal pattern that was the strongest predictor for CN vs. MCI classification in pre-ComBat analyses. Component 7 is characterized by low amyloid in occipital, parietal, brainstem, and cerebellar regions covarying with higher tau in the anterior temporal lobe and temporal pole, and lower tau in posterior association cortices (inferior parietal, precuneus, posterior cingulate, fusiform, and temporal gyri), linked to lower surface area in orbitofrontal and inferior parietal regions and greater surface area in frontal, temporal, and occipital areas. Upper row (left to right): cortical view for amyloid, tau, and pial surface area. Lower row (left to right): subcortical view for amyloid and tau. (B) Mean Component 7 loadings across diagnostic groups, stratified by *APOE4* carrier status. Error bars indicate 95% confidence intervals.

**References**

Coath, W., Modat, M., Cardoso, M. J., Markiewicz, P. J., Lane, C. A., Parker, T. D., … & Alzheimer’s Disease Neuroimaging Initiative. (2023). Operationalizing the centiloid scale for [¹⁸F] florbetapir PET studies on PET/MRI.  Alzheimer’s & Dementia: Diagnosis, Assessment & Disease Monitoring, 15(2), e12434.

Johnson, W. E., Li, C., & Rabinovic, A. (2007). Adjusting batch effects in microarray expression data using empirical Bayes methods.  Biostatistics, 8(1), 118–127.

Pomponio, R., Erus, G., Habes, M., Doshi, J., Srinivasan, D., Mamourian, E., … & Davatzikos, C. (2020). Harmonization of large MRI datasets for the analysis of brain imaging patterns throughout the lifespan.  NeuroImage, 208, 116450.

# D. Classification Models of Clinical Outcomes

For training the SBF loadings-based model and all comparator models, logistic regressions were fit with L1 (LASSO) regularization using the caret v7.0.1 interface to glmnet v4.1.9 package in R v4.3.2, tuning λ across a log-spaced grid from 1×10⁻³ to 1×10¹. Cross-validation was performed in a site-wise manner: participants from the same acquisition site were kept together, with 5 folds ensuring ~80% of sites for training and ~20% for validation in each fold, with all preprocessing (z-scoring) performed within each training fold to prevent information leakage. Performance on the independent test set was evaluated with the Area Under Receiver Operating Characteristic curve (AUROC; pROC package v1.18.5) and the Area Under the Precision–Recall (PR) Curve (AUPRC; PRROC package v1.4) in R v4.3.2, with 2,000 bootstrap confidence intervals. Class imbalance was handled using inverse-frequency sample weights (assigning half the total weight to the rarer class). Due to the limited size of the independent test set, threshold-dependent metrics (accuracy, sensitivity, specificity, precision, recall, F1 score, balanced accuracy) were derived from cross-validated predictions within the training set, using the threshold that maximized balanced accuracy. Cross-validated metrics are reported because the SBF loadings are derived from a single fully trained multimodal decomposition; recomputing SBF within each fold would re-estimate the latent multimodal space on much smaller subsets, leading to unstable and non-comparable representations across folds. Thus, cross validation evaluates the stability of the downstream classifier given a fixed, fully trained latent representation, rather than relearning the representation itself; whereas the fully independent (though smaller) test set provides an unbiased estimate of generalization. For multi-class outcomes (i.e., clinical diagnosis), one-versus-one (OvO) classification was conducted, and macro-averaged scores across all pairwise comparisons were reported.

# E. Comparator Models

Prediction performance of SBF loadings was compared against additional comparator model feature sets serving as reference points to assess whether SBF adds value over simpler or single-modality approaches, including: (1) demographics (age, sex, education); (2) single-modality imaging features derived from the top 50 principal components (PCs) of each raw modality—GM, CT, PSA, AMY and TAU; (3) a multimodal feature set composed of the top 10 PCs from each modality (50 total); (4) a simple “naïve” fusion approach in which PCA was applied to all modalities concatenated together, from which the top 50 PCs were extracted; and (5) independent component analysis (ICA) analogs of (2–4), in which 50 independent components were extracted in parallel to PCA comparator models. PCA was implemented using IncrementalPCA in Python 3.11.7 scikit-learn v 1.7.2, while ICA comparator models were generated with FastICA in scikit-learn.

# F. Study participants

The mean age was 70.8 years (SD = 6.9), and 56.2% (154/274) were female. Most were White (92.7%) and not Hispanic/Latino (96%). Overall, educational attainment was high: 44.2% held a graduate degree, 46% had some college or a college degree, and 9.9% had a high school education or less. *APOE4* carriers comprised 40.5% (111/274). Clinically, 63.9% were cognitively normal (CN), 21.9% had amnestic MCI, and 14.2% had dementia. By CDR-SOB, with a proposed translation (O’Bryant et al., 2008), 59.5% scored 0 (CN), 34.7% ranged 0.5–4.0 (MCI/SCC), and 5.8% ranged 4.5–9.0 (mild dementia). Missing data were minimal (≤ 5% across all variables, none for imaging, demographics, CDR-SOB, or diagnosis), with the exception of CSF measures, which had approximately 23% missingness. No significant group differences for demographic, cognitive measures (e.g., CDR-SOB p = 0.695, MMSE p = 0.753, ADAS-COG p = 0.828), or fluid biomarkers were observed across training, validation, and test sets (see Table 1). *APOE4* carrier prevalence was also comparable across splits (χ² p = 0.732; Cramér’s V = 0.048), with no evidence of enrichment across imaging sites (Monte Carlo Fisher p = 0.133). Additionally, using the precomputed ADNI amyloid status variable, amyloid positivity rates were 25.7% in CN (45/175), 45.0% in MCI (27/60), and 82.1% in mild dementia (32/39).

Reference:

O’Bryant SE, Waring SC, Cullum CM, Hall J, Lacritz L, Massman PJ, et al. Staging Dementia Using Clinical Dementia Rating Scale Sum of Boxes Scores: A Texas Alzheimer’s Research Consortium Study. Arch Neurol 2008;65:1091. https://doi.org/10.1001/ARCHNEUR.65.8.1091.

# G. Sensitivity of CDR-SOB Prediction to Dimensionality and Random Initialization

To evaluate the sensitivity of the continuous CDR-SOB prediction to the choice of model dimensionality and random initialization, we repeated the full SBF pipeline across 9 dimensionality configurations (10, 15, 20, 25, 30, 35, 40, 45, and 50 latent components) with 5 random initialization rounds for each configuration (45 models total). For each model, the Pearson correlation between predicted and observed CDR-SOB scores was computed in the independent test set.

As shown in Figure S3, CDR-SOB prediction performance was broadly stable across dimensionalities, with mean correlations per dimension ranging from approximately 0.25 to 0.32. No systematic trend was observed as model order increased (Pearson r = −0.29 between model order and average performance, p = 0.441; no significant main effect of dimensionality, F = 0.31, p = 0.526), suggesting that the primary 50-component model does not represent an outlier configuration. These results support the robustness of SBF's continuous prediction performance to the choice of latent dimensionality.


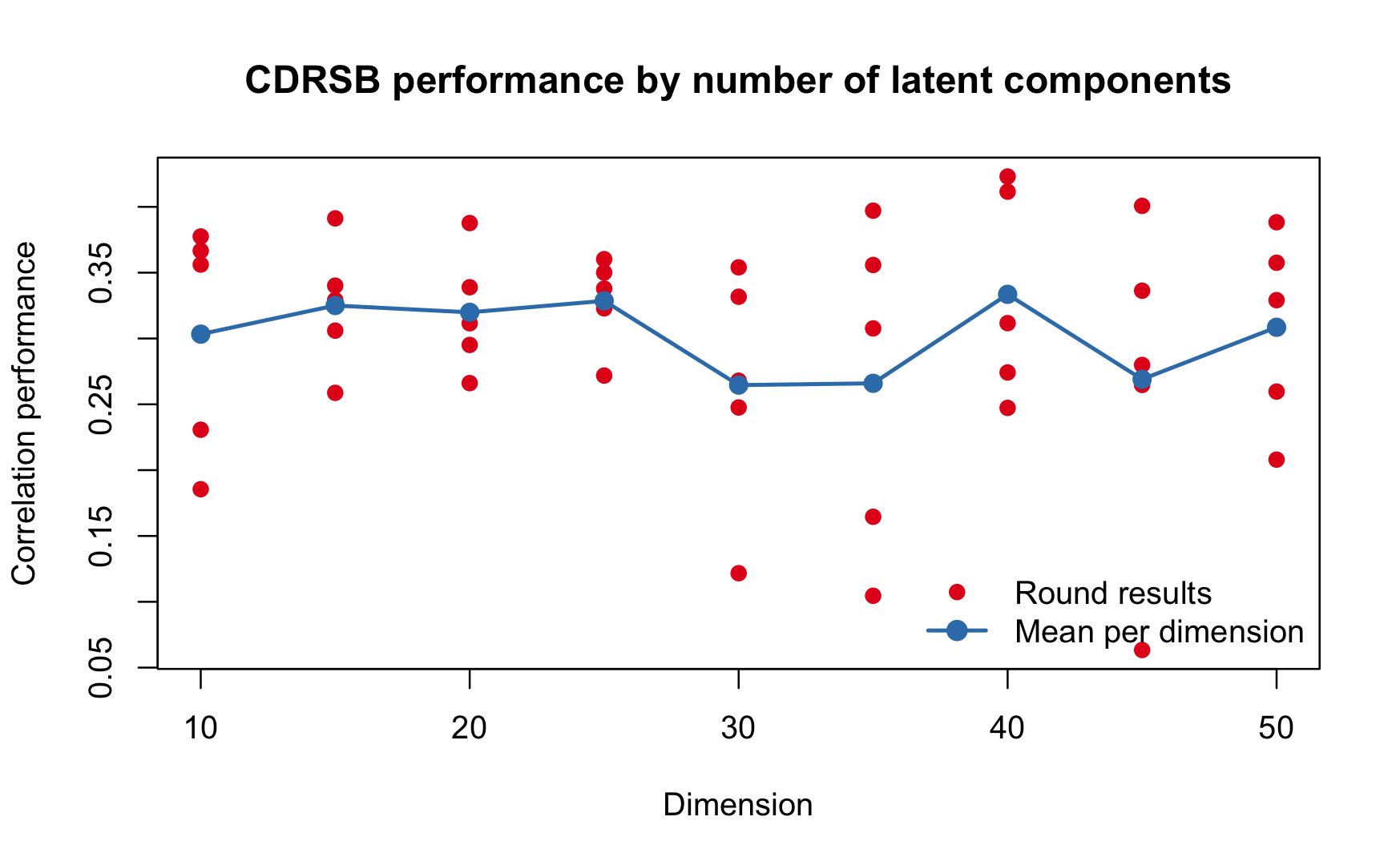


### Figure S3. CDR-SOB prediction by dimensionality and random initialization

**Figure S3.** *CDR-SOB prediction performance (Pearson correlation) by number of latent components across 5 random initialization rounds.* Red dots represent individual round results; blue line with dots shows the mean correlation per dimensionality. Performance was broadly stable across the 9 dimensionality configurations (10–50 components, step 5).

# H. Classification Tables and Figures


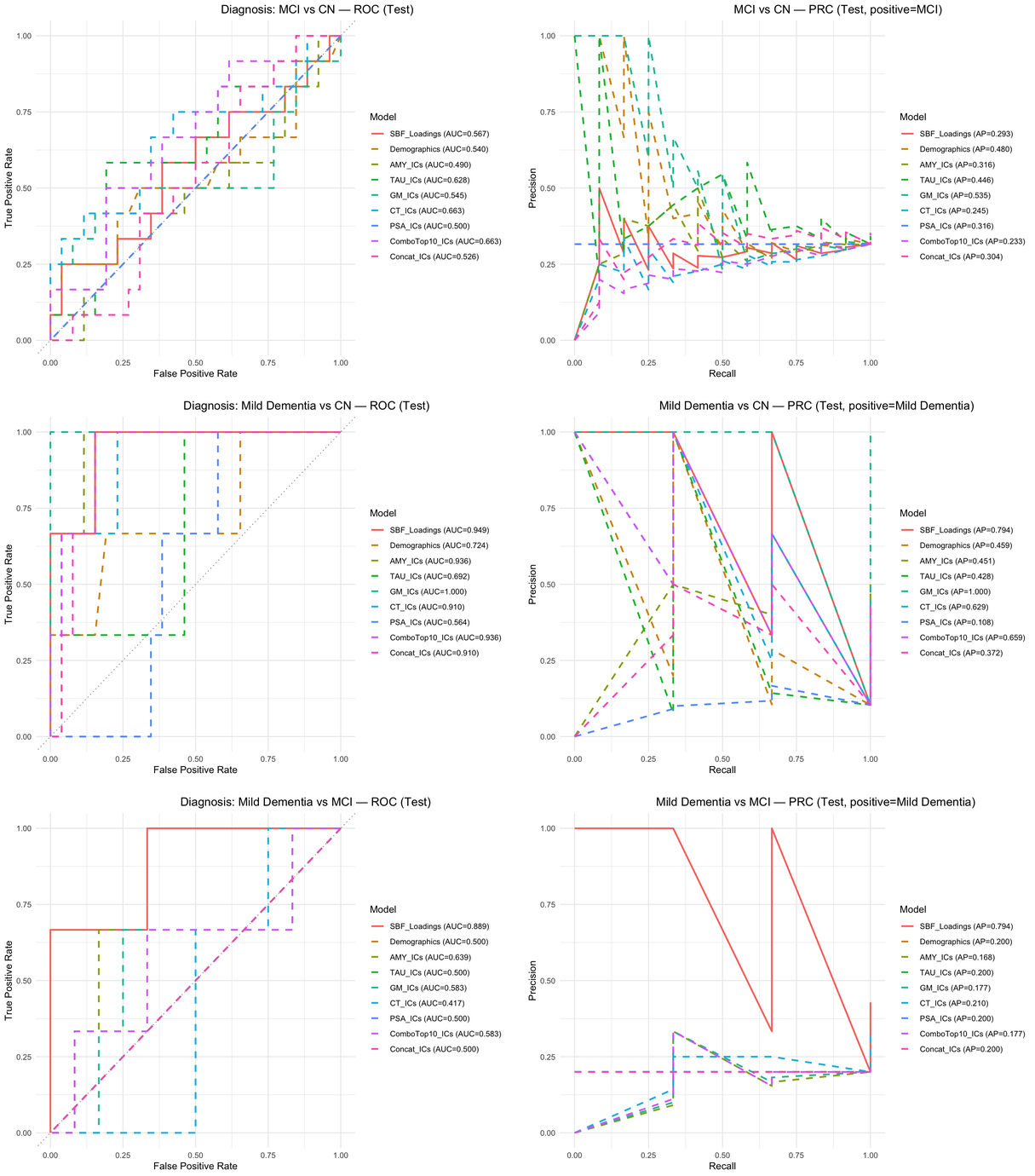


### Figure S4. Diagnosis prediction: SBF, demographics, and ICA models

**Figure S4. Model performance in predicting clinical diagnoses, comparing SBF loadings–based models, demographic comparator model, and ICA comparator models.**

A. ROC curve for CN vs. MCI. B. PRC curve for CN vs. MCI. C. ROC curve for CN vs. dementia. D. PRC curve for CN vs. dementia. E. ROC curve for MCI vs. dementia. F. PRC curve for MCI vs. dementia. The analyses indicated that, compared with all comparator models, the model based on SBF loadings strong performance for dementia discrimination for CN vs. mild dementia and MCI vs. mild dementia, with weaker separation of CN vs. MCI. AUPRC exhibited the same relative performance profile, confirming that the observed discriminative ranking was not driven by test set imbalance.


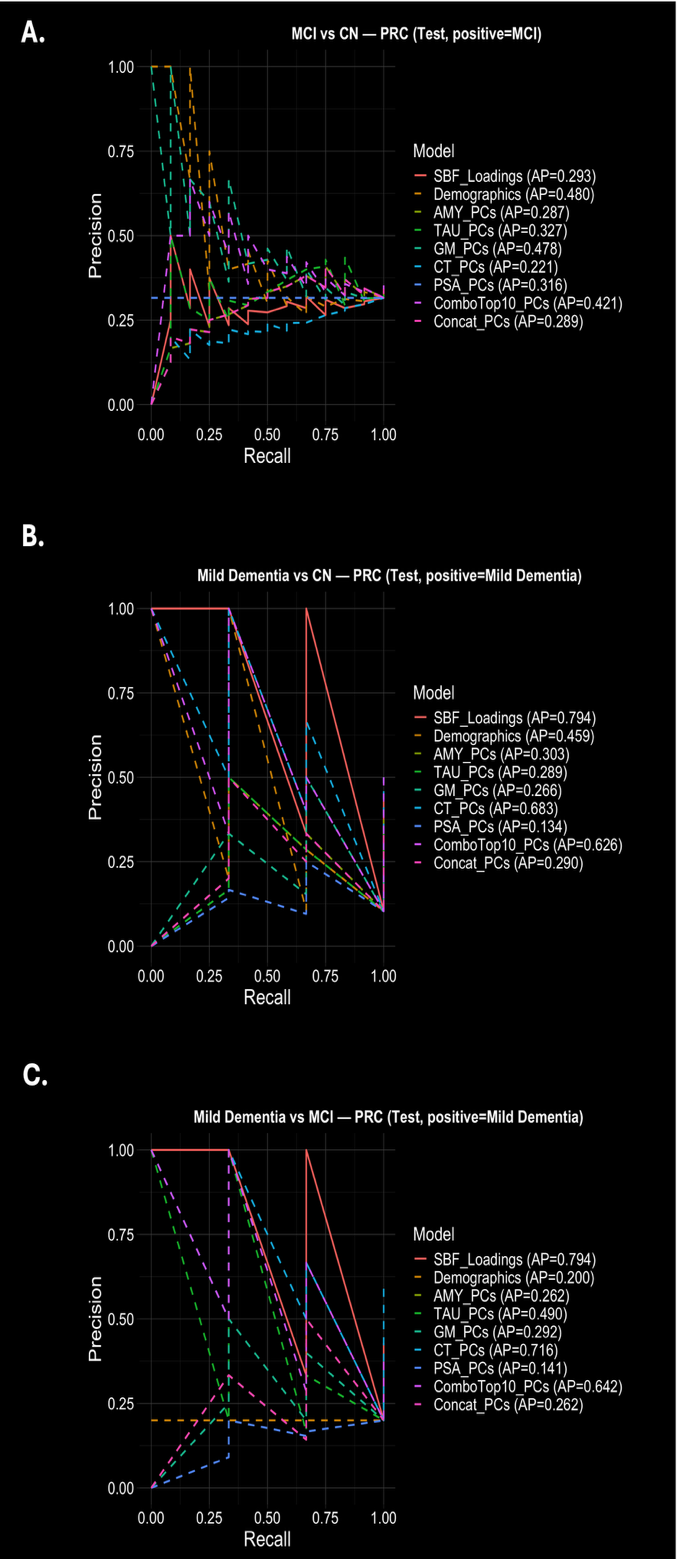


### Figure S5. Diagnosis prediction: PCA-based feature loadings

**Figure S5. Model performance in predicting clinical diagnoses using PCA-based feature loadings (post-ComBat).**

A. PRC curve for CN vs. MCI. B. PRC curve for CN vs. Mild Dementia. C. PRC curve for MCI vs. Mild Dementia. Results show post-ComBat performance comparing SBF loadings against PCA-based unimodal and multimodal comparator models.


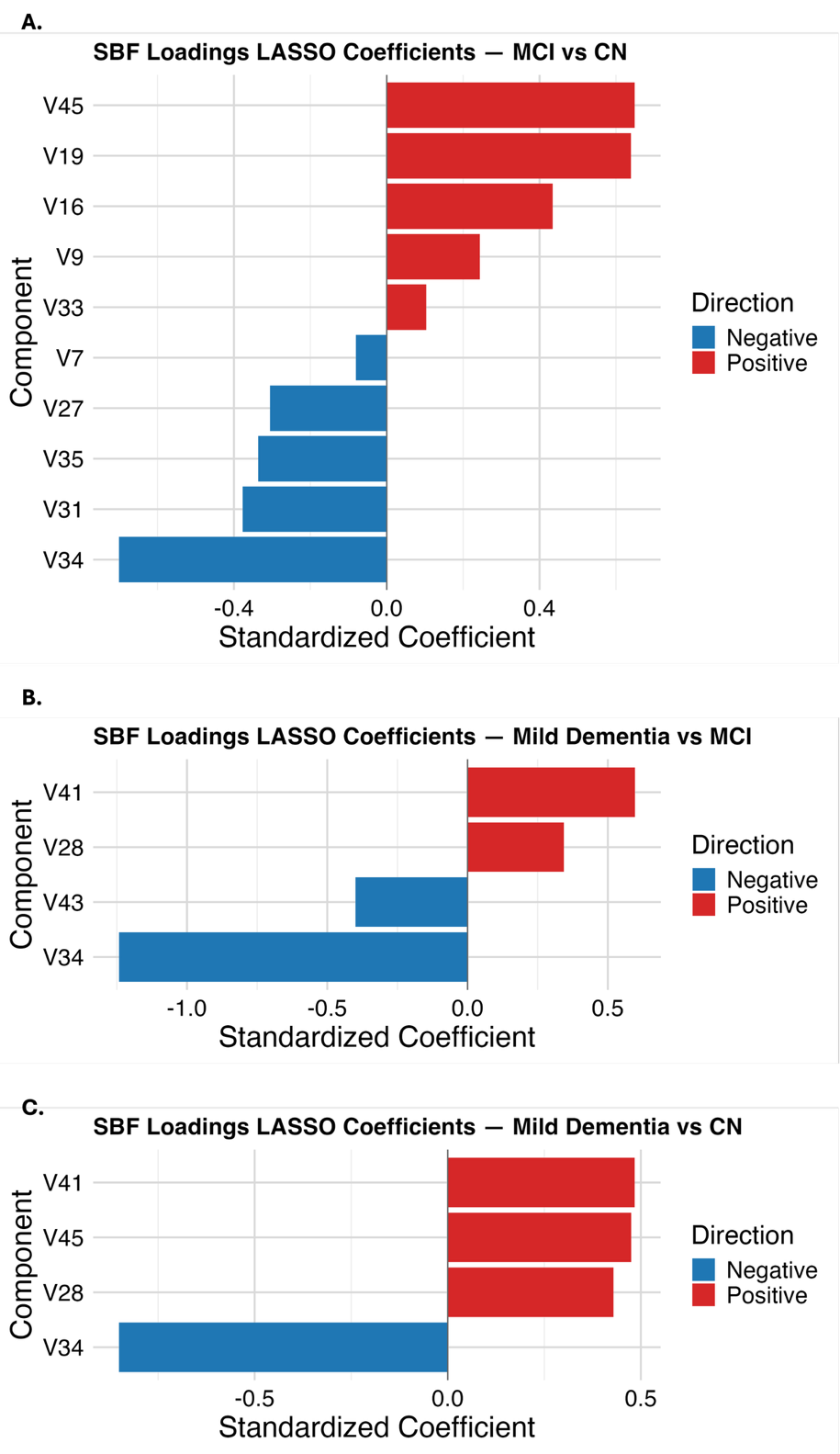


### Figure S6. Feature importance for diagnosis classification

**Figure S6. LASSO-selected SuperBigFLICA (SBF) loading coefficients for pairwise diagnosis classification.** Horizontal bar charts show the standardized coefficients of SBF latent component loadings selected by LASSO-penalized logistic regression (L1 regularization) for each one-vs-one diagnosis comparison: (A) MCI vs. cognitively normal (CN), (B) mild dementia vs. MCI, and (C) mild dementia vs. CN. Only components with non-zero coefficients at the cross-validation-selected regularization parameter (λ) are displayed. All features were standardized (zero mean, unit variance) prior to model fitting. Models were trained on ComBat-harmonized SBF loadings with site-grouped cross-validation.


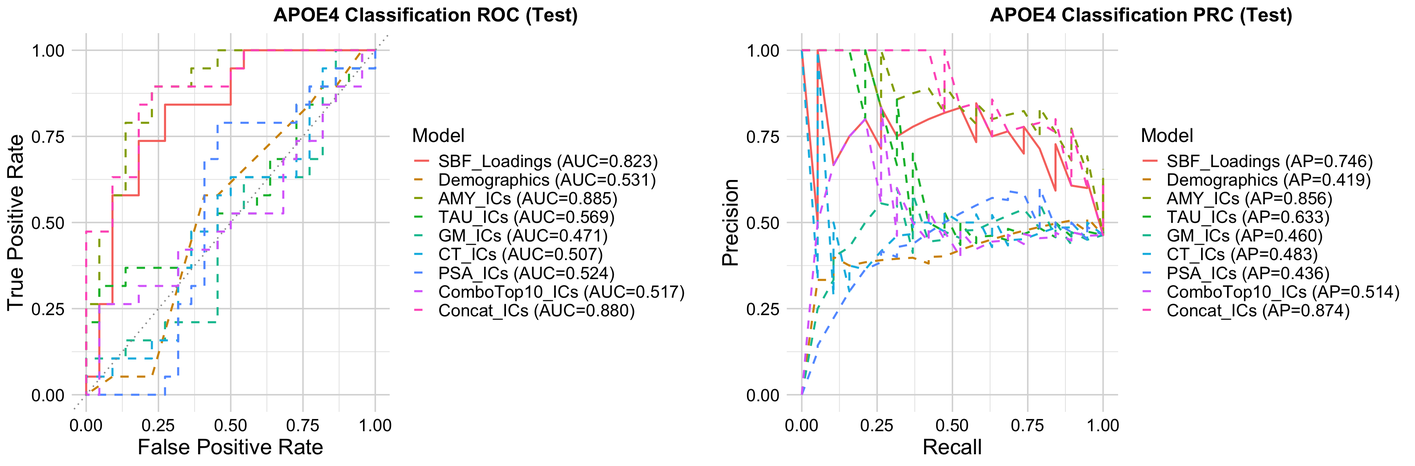


### Figure S7. APOE4 prediction: SBF, demographics, and ICA models

**Figure S7.** **Model performance in predicting *APOE4* status, comparing SBF loadings–based models, demographic comparator model, and ICA comparator models.**

A. ROC curve for *APOE4* carrier vs. non-carrier. B. PRC curve for *APOE4* carrier vs. non-carrier. The results showed the model based on SBF loadings had stronger performance for *APOE4* carrier/non-carrier discrimination than all comparator models.

### Table S12. Diagnosis classification: SBF vs. ICA comparators (macro-averaged)

**Table S12. Model performance in predicting clinical diagnoses, comparing SBF loadings–based models, demographic comparator model, and ICA comparator models.**

| **Model** | **AUROC (test)** | **AUPRC (test)** | **Accuracy (CV)** | **Balanced Accuracy (CV)** | **Sensitivity (CV)** | **Specificity (CV)** | **Precision (CV)** | **F1 (CV)** |
| --- | --- | --- | --- | --- | --- | --- | --- | --- |
| SBF Loadings | **0.80 [0.59, 0.92]** | **0.68 [0.14, 0.89]** | **0.85 [0.78, 0.91]** | **0.81 [0.73, 0.88]** | 0.73 [0.57, 0.85] | **0.90 [0.82, 0.95]** | **0.70 [0.55, 0.84]** | **0.71 [0.57, 0.81]** |
| Demographics | 0.59 [0.46, 0.76] | 0.38 [0.10, 0.71] | 0.57 [0.48, 0.66] | 0.63 [0.57, 0.68] | 0.85 [0.75, 0.94] | 0.40 [0.34, 0.46] | 0.37 [0.26, 0.48] | 0.51 [0.39, 0.63] |
| Amyloid ICs | 0.69 [0.55, 0.92] | 0.36 [0.11, 0.86] | 0.62 [0.53, 0.71] | 0.64 [0.54, 0.73] | 0.73 [0.58, 0.89] | 0.54 [0.44, 0.64] | 0.39 [0.27, 0.52] | 0.51 [0.38, 0.63] |
| Tau ICs | 0.61 [0.44, 0.78] | 0.36 [0.10, 0.71] | 0.64 [0.55, 0.73] | 0.58 [0.52, 0.64] | 0.62 [0.51, 0.73] | 0.55 [0.50, 0.58] | 0.44 [0.28, 0.60] | 0.47 [0.32, 0.60] |
| Grey Matter ICs | 0.71 [0.62, 0.90] | 0.59 [0.43, 0.86] | 0.75 [0.66, 0.83] | 0.66 [0.56, 0.75] | 0.43 [0.27, 0.59] | 0.88 [0.81, 0.94] | 0.56 [0.34, 0.78] | 0.45 [0.27, 0.60] |
| Cortical Thickness ICs | 0.66 [0.53, 0.90] | 0.46 [0.15, 0.74] | 0.68 [0.60, 0.76] | 0.63 [0.54, 0.70] | 0.64 [0.50, 0.76] | 0.61 [0.54, 0.69] | 0.45 [0.30, 0.59] | 0.51 [0.36, 0.63] |
| Pial Surface Area ICs | 0.52 [0.49, 0.60] | 0.21 [0.09, 0.38] | 0.40 [0.31, 0.49] | 0.52 [0.48, 0.56] | **0.86 [0.79, 0.92]** | 0.18 [0.15, 0.22] | 0.29 [0.20, 0.38] | 0.43 [0.32, 0.52] |
| Top 10 ICs Combo | 0.73 [0.56, 0.94] | 0.48 [0.12, 0.88] | 0.55 [0.46, 0.64] | 0.61 [0.52, 0.69] | 0.76 [0.59, 0.89] | 0.46 [0.35, 0.57] | 0.35 [0.24, 0.45] | 0.47 [0.35, 0.58] |
| Concatenated ICs | 0.65 [0.57, 0.74] | 0.29 [0.11, 0.65] | 0.63 [0.54, 0.71] | 0.62 [0.56, 0.68] | 0.73 [0.62, 0.83] | 0.51 [0.46, 0.56] | 0.40 [0.26, 0.52] | 0.51 [0.37, 0.62] |

All metrics are reported as macro-averages across pairwise comparisons (CN vs. MCI, CN vs. dementia, MCI vs. dementia). Values are shown with 95% confidence intervals on the second line of each cell. The best performance under each metric is bolded. CV means the result was from cross-validated training data.

### Table S13. CN vs. MCI: SBF vs. ICA comparators

**Table S13. Pairwise classification performance: CN vs. MCI, comparing SBF loadings–based models, demographic comparator model, and ICA comparator models.**

| **Model** | **AUROC (test)** | **AUPRC (test)** | **Accuracy (CV)** | **Balanced Accuracy (CV)** | **Sensitivity (CV)** | **Specificity (CV)** | **Precision (CV)** | **F1 (CV)** |
| --- | --- | --- | --- | --- | --- | --- | --- | --- |
| SBF Loadings | 0.57 [0.45, 0.76] | 0.45 [0.19, 0.68] | **0.80 [0.74, 0.86]** | **0.71 [0.63, 0.79]** | 0.54 [0.39, 0.68] | 0.88 [0.82, 0.93] | **0.57 [0.41, 0.72]** | **0.56 [0.40, 0.68]** |
| Demographics | 0.54 [0.45, 0.77] | 0.48 [0.19, 0.72] | 0.63 [0.55, 0.70] | 0.66 [0.57, 0.73] | 0.70 [0.55, 0.84] | 0.61 [0.52, 0.69] | 0.35 [0.24, 0.45] | 0.46 [0.34, 0.57] |
| Amyloid ICs | 0.49 [0.43, 0.76] | 0.32 [0.16, 0.57] | 0.60 [0.53, 0.67] | 0.60 [0.51, 0.69] | 0.59 [0.44, 0.76] | 0.60 [0.52, 0.69] | 0.30 [0.20, 0.42] | 0.40 [0.28, 0.52] |
| Tau ICs | 0.63 [0.44, 0.83] | 0.45 [0.21, 0.72] | 0.66 [0.58, 0.73] | 0.63 [0.54, 0.72] | 0.57 [0.41, 0.73] | 0.69 [0.61, 0.77] | 0.35 [0.23, 0.47] | 0.43 [0.30, 0.54] |
| Grey Matter ICs | 0.54 [0.44, 0.78] | 0.54 [0.26, 0.77] | 0.77 [0.71, 0.84] | 0.57 [0.51, 0.64] | 0.19 [0.08, 0.32] | **0.94 [0.91, 0.98]** | 0.50 [0.25, 0.78] | 0.27 [0.12, 0.44] |
| Cortical Thickness ICs | **0.66 [0.45, 0.85]** | **0.59 [0.32, 0.82]** | 0.74 [0.68, 0.80] | 0.60 [0.51, 0.68] | 0.35 [0.20, 0.50] | 0.85 [0.79, 0.91] | 0.41 [0.25, 0.57] | 0.38 [0.22, 0.51] |
| Pial Surface Area ICs | 0.50 [0.50, 0.50] | 0.32 [0.18, 0.47] | 0.24 [0.17, 0.30] | 0.51 [0.50, 0.52] | **1.00 [1.00, 1.00]** | 0.02 [0.00, 0.04] | 0.23 [0.16, 0.30] | 0.37 [0.28, 0.46] |
| Top 10 ICs Combo | **0.66 [0.46, 0.83]** | 0.51 [0.22, 0.73] | 0.49 [0.41, 0.57] | 0.56 [0.46, 0.64] | 0.68 [0.51, 0.81] | 0.44 [0.35, 0.52] | 0.26 [0.18, 0.34] | 0.38 [0.27, 0.46] |
| Concatenated ICs | 0.53 [0.45, 0.72] | 0.30 [0.16, 0.54] | 0.70 [0.63, 0.77] | 0.60 [0.50, 0.69] | 0.41 [0.24, 0.57] | 0.79 [0.72, 0.86] | 0.36 [0.21, 0.50] | 0.38 [0.23, 0.51] |

All threshold-free metrics (AUROC, AUPRC) are reported on the held-out test set; threshold-based metrics (Accuracy through F1) are from cross-validation. Values are shown with 95% bootstrap confidence intervals. The best performance under each metric is bolded. CV = cross-validated training data.

### Table S14. CN vs. Mild Dementia: SBF vs. ICA comparators

**Table S14. Pairwise classification performance: CN vs. Mild Dementia, comparing SBF loadings–based models, demographic comparator model, and ICA comparator models.**

| **Model** | **AUROC (test)** | **AUPRC (test)** | **Accuracy (CV)** | **Balanced Accuracy (CV)** | **Sensitivity (CV)** | **Specificity (CV)** | **Precision (CV)** | **F1 (CV)** |
| --- | --- | --- | --- | --- | --- | --- | --- | --- |
| SBF Loadings | 0.95 [0.79, 1.00] | 0.79 [0.11, 1.00] | **0.91 [0.86, 0.95]** | **0.90 [0.83, 0.96]** | **0.89 [0.75, 1.00]** | 0.91 [0.86, 0.95] | **0.69 [0.54, 0.84]** | **0.78 [0.65, 0.88]** |
| Demographics | 0.72 [0.42, 1.00] | 0.46 [0.03, 1.00] | 0.65 [0.57, 0.72] | 0.73 [0.64, 0.81] | 0.86 [0.70, 0.97] | 0.60 [0.51, 0.69] | 0.32 [0.22, 0.43] | 0.47 [0.35, 0.58] |
| Amyloid ICs | 0.94 [0.81, 1.00] | 0.45 [0.14, 1.00] | 0.77 [0.69, 0.84] | 0.76 [0.67, 0.85] | 0.75 [0.60, 0.93] | 0.77 [0.69, 0.84] | 0.42 [0.29, 0.55] | 0.54 [0.40, 0.67] |
| Tau ICs | 0.69 [0.38, 1.00] | 0.43 [0.03, 1.00] | 0.83 [0.76, 0.88] | 0.62 [0.54, 0.70] | 0.29 [0.13, 0.45] | **0.94 [0.90, 0.98]** | 0.53 [0.31, 0.75] | 0.37 [0.19, 0.54] |
| Grey Matter ICs | **1.00 [1.00, 1.00]** | **1.00 [1.00, 1.00]** | 0.79 [0.73, 0.86] | 0.76 [0.66, 0.85] | 0.71 [0.54, 0.87] | 0.81 [0.74, 0.88] | 0.45 [0.31, 0.61] | 0.56 [0.41, 0.68] |
| Cortical Thickness ICs | 0.91 [0.74, 1.00] | 0.63 [0.08, 1.00] | 0.81 [0.74, 0.87] | 0.73 [0.63, 0.82] | 0.61 [0.42, 0.79] | 0.85 [0.79, 0.91] | 0.47 [0.32, 0.63] | 0.53 [0.37, 0.66] |
| Pial Surface Area ICs | 0.56 [0.47, 0.79] | 0.11 [0.03, 0.26] | 0.54 [0.46, 0.61] | 0.55 [0.45, 0.65] | 0.57 [0.38, 0.76] | 0.53 [0.44, 0.62] | 0.21 [0.13, 0.29] | 0.31 [0.20, 0.41] |
| Top 10 ICs Combo | 0.94 [0.79, 1.00] | 0.66 [0.11, 1.00] | 0.63 [0.56, 0.71] | 0.71 [0.62, 0.78] | 0.82 [0.67, 0.95] | 0.59 [0.50, 0.68] | 0.31 [0.21, 0.40] | 0.45 [0.33, 0.55] |
| Concatenated ICs | 0.91 [0.76, 1.00] | 0.37 [0.11, 1.00] | 0.75 [0.68, 0.82] | 0.77 [0.68, 0.85] | 0.79 [0.63, 0.93] | 0.75 [0.67, 0.82] | 0.41 [0.27, 0.53] | 0.54 [0.39, 0.66] |

All threshold-free metrics (AUROC, AUPRC) are reported on the held-out test set; threshold-based metrics (Accuracy through F1) are from cross-validation. Values are shown with 95% bootstrap confidence intervals. The best performance under each metric is bolded. CV = cross-validated training data.

### Table S15. MCI vs. Mild Dementia: SBF vs. ICA comparators

**Table S15. Pairwise classification performance: MCI vs. Mild Dementia, comparing SBF loadings–based models, demographic comparator model, and ICA comparator models.**

| **Model** | **AUROC (test)** | **AUPRC (test)** | **Accuracy (CV)** | **Balanced Accuracy (CV)** | **Sensitivity (CV)** | **Specificity (CV)** | **Precision (CV)** | **F1 (CV)** |
| --- | --- | --- | --- | --- | --- | --- | --- | --- |
| SBF Loadings | **0.89 [0.54, 1.00]** | **0.79 [0.11, 1.00]** | **0.83 [0.72, 0.91]** | **0.82 [0.72, 0.90]** | 0.75 [0.57, 0.88] | **0.89 [0.78, 0.97]** | **0.84 [0.70, 0.96]** | **0.79 [0.65, 0.89]** |
| Demographics | 0.50 [0.50, 0.50] | 0.20 [0.07, 0.40] | 0.43 [0.31, 0.57] | 0.50 [0.50, 0.50] | **1.00 [1.00, 1.00]** | 0.00 [0.00, 0.00] | 0.43 [0.31, 0.57] | 0.60 [0.47, 0.73] |
| Amyloid ICs | 0.64 [0.42, 1.00] | 0.31 [0.04, 1.00] | 0.51 [0.38, 0.62] | 0.55 [0.45, 0.64] | 0.86 [0.71, 0.97] | 0.24 [0.11, 0.39] | 0.46 [0.33, 0.59] | 0.60 [0.46, 0.71] |
| Tau ICs | 0.50 [0.50, 0.50] | 0.20 [0.07, 0.40] | 0.43 [0.31, 0.57] | 0.50 [0.50, 0.50] | **1.00 [1.00, 1.00]** | 0.00 [0.00, 0.00] | 0.43 [0.31, 0.57] | 0.60 [0.47, 0.73] |
| Grey Matter ICs | 0.58 [0.42, 0.93] | 0.24 [0.04, 0.83] | 0.68 [0.55, 0.78] | 0.64 [0.52, 0.75] | 0.39 [0.20, 0.58] | **0.89 [0.78, 0.98]** | 0.73 [0.46, 0.94] | 0.51 [0.28, 0.68] |
| Cortical Thickness ICs | 0.42 [0.41, 0.86] | 0.16 [0.04, 0.41] | 0.49 [0.37, 0.61] | 0.55 [0.49, 0.61] | 0.96 [0.88, 1.00] | 0.14 [0.05, 0.25] | 0.46 [0.32, 0.58] | 0.62 [0.49, 0.73] |
| Pial Surface Area ICs | 0.50 [0.50, 0.50] | 0.20 [0.07, 0.40] | 0.43 [0.31, 0.54] | 0.50 [0.50, 0.50] | **1.00 [1.00, 1.00]** | 0.00 [0.00, 0.00] | 0.43 [0.31, 0.54] | 0.60 [0.47, 0.70] |
| Top 10 ICs Combo | 0.58 [0.44, 1.00] | 0.26 [0.04, 0.91] | 0.54 [0.41, 0.65] | 0.57 [0.46, 0.67] | 0.79 [0.60, 0.91] | 0.35 [0.19, 0.49] | 0.48 [0.33, 0.61] | 0.59 [0.44, 0.71] |
| Concatenated ICs | 0.50 [0.50, 0.50] | 0.20 [0.07, 0.40] | 0.43 [0.31, 0.54] | 0.50 [0.50, 0.50] | **1.00 [1.00, 1.00]** | 0.00 [0.00, 0.00] | 0.43 [0.31, 0.54] | 0.60 [0.47, 0.70] |

All threshold-free metrics (AUROC, AUPRC) are reported on the held-out test set; threshold-based metrics (Accuracy through F1) are from cross-validation. Values are shown with 95% bootstrap confidence intervals. The best performance under each metric is bolded. CV = cross-validated training data.

### Table S16. CN vs. MCI: SBF vs. PCA comparators

**Table S16. Pairwise classification performance: CN vs. MCI, comparing SBF loadings–based models, demographic comparator model, and PCA comparator models. Best metric is bolded. CV = cross-validated training data.**

| **Model** | **AUROC (test)** | **AUPRC (test)** | **Accuracy (CV)** | **Balanced Accuracy (CV)** | **Sensitivity (CV)** | **Specificity (CV)** | **Precision (CV)** | **F1 (CV)** |
| --- | --- | --- | --- | --- | --- | --- | --- | --- |
| SBF Loadings | 0.57 [0.45, 0.76] | 0.29 [0.15, 0.56] | **0.80 [0.74, 0.86]** | **0.71 [0.63, 0.79]** | 0.54 [0.39, 0.68] | 0.88 [0.82, 0.93] | 0.57 [0.41, 0.72] | **0.56 [0.40, 0.68]** |
| Demographics | 0.54 [0.45, 0.77] | **0.48 [0.19, 0.72]** | 0.63 [0.55, 0.70] | 0.66 [0.57, 0.73] | 0.70 [0.55, 0.84] | 0.61 [0.52, 0.69] | 0.35 [0.24, 0.45] | 0.46 [0.34, 0.57] |
| Amyloid PCs | 0.50 [0.44, 0.72] | 0.29 [0.15, 0.48] | 0.71 [0.64, 0.77] | 0.63 [0.54, 0.71] | 0.49 [0.34, 0.64] | 0.77 [0.70, 0.84] | 0.38 [0.26, 0.52] | 0.43 [0.30, 0.55] |
| Tau PCs | 0.54 [0.40, 0.73] | 0.33 [0.18, 0.58] | 0.75 [0.67, 0.81] | 0.66 [0.56, 0.75] | 0.49 [0.31, 0.66] | 0.83 [0.75, 0.89] | 0.45 [0.27, 0.60] | 0.47 [0.30, 0.60] |
| Grey Matter PCs | 0.60 [0.47, 0.81] | **0.48 [0.22, 0.73]** | **0.80 [0.74, 0.87]** | 0.66 [0.59, 0.74] | 0.41 [0.25, 0.56] | **0.92 [0.87, 0.96]** | **0.60 [0.40, 0.79]** | 0.48 [0.32, 0.62] |
| Cortical Thickness PCs | **0.72 [0.45, 0.88]** | 0.22 [0.11, 0.38] | 0.77 [0.70, 0.84] | 0.58 [0.51, 0.67] | 0.24 [0.11, 0.40] | **0.92 [0.87, 0.96]** | 0.47 [0.26, 0.72] | 0.32 [0.16, 0.49] |
| Pial Surface Area PCs | 0.50 [0.50, 0.50] | 0.32 [0.18, 0.47] | 0.23 [0.16, 0.28] | 0.50 [0.50, 0.50] | **1.00 [1.00, 1.00]** | 0.00 [0.00, 0.00] | 0.23 [0.16, 0.28] | 0.37 [0.28, 0.44] |
| Top 10 PCs Combo | 0.62 [0.45, 0.81] | 0.42 [0.21, 0.71] | 0.79 [0.74, 0.84] | 0.67 [0.59, 0.75] | 0.46 [0.30, 0.61] | 0.89 [0.84, 0.93] | 0.55 [0.37, 0.70] | 0.50 [0.35, 0.63] |
| Concatenated PCs | 0.51 [0.44, 0.72] | 0.29 [0.15, 0.49] | 0.70 [0.62, 0.77] | 0.63 [0.54, 0.72] | 0.51 [0.36, 0.66] | 0.75 [0.68, 0.82] | 0.37 [0.25, 0.51] | 0.43 [0.31, 0.55] |

### Table S17. CN vs. Mild Dementia: SBF vs. PCA comparators

**Table S17. Pairwise classification performance: CN vs. Mild Dementia, comparing SBF loadings–based models, demographic comparator model, and PCA comparator models. Best metric is bolded. CV = cross-validated training data.**

| **Model** | **AUROC (test)** | **AUPRC (test)** | **Accuracy (CV)** | **Balanced Accuracy (CV)** | **Sensitivity (CV)** | **Specificity (CV)** | **Precision (CV)** | **F1 (CV)** |
| --- | --- | --- | --- | --- | --- | --- | --- | --- |
| SBF Loadings | **0.95 [0.79, 1.00]** | **0.79 [0.11, 1.00]** | **0.91 [0.86, 0.95]** | 0.90 [0.83, 0.96] | 0.89 [0.75, 1.00] | 0.91 [0.86, 0.95] | 0.69 [0.54, 0.84] | 0.78 [0.65, 0.88] |
| Demographics | 0.72 [0.42, 1.00] | 0.46 [0.03, 1.00] | 0.65 [0.57, 0.72] | 0.73 [0.64, 0.81] | 0.86 [0.70, 0.97] | 0.60 [0.51, 0.69] | 0.32 [0.22, 0.43] | 0.47 [0.35, 0.58] |
| Amyloid PCs | 0.87 [0.70, 1.00] | 0.30 [0.09, 0.99] | 0.89 [0.85, 0.94] | 0.84 [0.75, 0.92] | 0.75 [0.58, 0.90] | 0.92 [0.88, 0.97] | 0.68 [0.50, 0.84] | 0.71 [0.56, 0.83] |
| Tau PCs | 0.86 [0.67, 1.00] | 0.29 [0.08, 1.00] | 0.90 [0.85, 0.94] | 0.87 [0.79, 0.93] | 0.82 [0.67, 0.96] | 0.91 [0.86, 0.96] | 0.68 [0.52, 0.83] | 0.74 [0.62, 0.86] |
| Grey Matter PCs | 0.81 [0.52, 1.00] | 0.27 [0.04, 1.00] | 0.79 [0.72, 0.85] | 0.83 [0.75, 0.89] | 0.89 [0.75, 1.00] | 0.77 [0.69, 0.84] | 0.46 [0.34, 0.59] | 0.61 [0.48, 0.71] |
| Cortical Thickness PCs | **0.95 [0.85, 1.00]** | 0.68 [0.14, 1.00] | 0.86 [0.81, 0.92] | 0.88 [0.80, 0.93] | 0.89 [0.76, 1.00] | 0.86 [0.80, 0.92] | 0.58 [0.42, 0.73] | 0.70 [0.56, 0.81] |
| Pial Surface Area PCs | 0.62 [0.44, 0.92] | 0.13 [0.02, 0.42] | 0.70 [0.61, 0.76] | 0.66 [0.55, 0.75] | 0.61 [0.42, 0.77] | 0.72 [0.63, 0.79] | 0.32 [0.20, 0.44] | 0.42 [0.28, 0.54] |
| Top 10 PCs Combo | 0.94 [0.80, 1.00] | 0.63 [0.14, 1.00] | **0.91 [0.86, 0.95]** | **0.94 [0.92, 0.97]** | **1.00 [1.00, 1.00]** | 0.89 [0.83, 0.94] | 0.67 [0.52, 0.82] | **0.80 [0.68, 0.90]** |
| Concatenated PCs | 0.86 [0.67, 1.00] | 0.29 [0.07, 0.99] | 0.90 [0.85, 0.94] | 0.84 [0.75, 0.92] | 0.75 [0.58, 0.90] | **0.93 [0.89, 0.97]** | **0.70 [0.52, 0.86]** | 0.72 [0.57, 0.84] |

### Table S18. MCI vs. Mild Dementia: SBF vs. PCA comparators

**Table S18. Pairwise classification performance: MCI vs. Mild Dementia, comparing SBF loadings–based models, demographic comparator model, and PCA comparator models. Best metric is bolded. CV = cross-validated training data.**

| **Model** | **AUROC (test)** | **AUPRC (test)** | **Accuracy (CV)** | **Balanced Accuracy (CV)** | **Sensitivity (CV)** | **Specificity (CV)** | **Precision (CV)** | **F1 (CV)** |
| --- | --- | --- | --- | --- | --- | --- | --- | --- |
| SBF Loadings | 0.89 [0.54, 1.00] | **0.79 [0.11, 1.00]** | 0.83 [0.72, 0.91] | 0.82 [0.72, 0.90] | 0.75 [0.57, 0.88] | 0.89 [0.78, 0.97] | 0.84 [0.70, 0.96] | 0.79 [0.65, 0.89] |
| Demographics | 0.50 [0.50, 0.50] | 0.20 [0.07, 0.40] | 0.43 [0.31, 0.57] | 0.50 [0.50, 0.50] | **1.00 [1.00, 1.00]** | 0.00 [0.00, 0.00] | 0.43 [0.31, 0.57] | 0.60 [0.47, 0.73] |
| Amyloid PCs | 0.56 [0.39, 1.00] | 0.26 [0.03, 0.92] | 0.71 [0.58, 0.82] | 0.70 [0.58, 0.81] | 0.64 [0.46, 0.80] | 0.76 [0.61, 0.88] | 0.67 [0.48, 0.83] | 0.65 [0.49, 0.77] |
| Tau PCs | 0.61 [0.46, 1.00] | 0.49 [0.04, 1.00] | 0.69 [0.58, 0.80] | 0.65 [0.56, 0.75] | 0.36 [0.19, 0.54] | 0.95 [0.86, 1.00] | 0.83 [0.61, 1.00] | 0.50 [0.30, 0.68] |
| Grey Matter PCs | 0.67 [0.42, 1.00] | 0.29 [0.05, 1.00] | 0.75 [0.65, 0.85] | 0.72 [0.62, 0.82] | 0.46 [0.28, 0.65] | **0.97 [0.91, 1.00]** | **0.93 [0.75, 1.00]** | 0.62 [0.41, 0.78] |
| Cortical Thickness PCs | **0.92 [0.72, 1.00]** | 0.72 [0.19, 1.00] | 0.78 [0.68, 0.88] | 0.78 [0.66, 0.88] | 0.71 [0.54, 0.89] | 0.84 [0.71, 0.95] | 0.77 [0.59, 0.93] | 0.74 [0.58, 0.86] |
| Pial Surface Area PCs | 0.69 [0.42, 1.00] | 0.14 [0.04, 0.39] | 0.55 [0.42, 0.68] | 0.60 [0.49, 0.69] | 0.89 [0.76, 1.00] | 0.30 [0.16, 0.45] | 0.49 [0.36, 0.62] | 0.63 [0.50, 0.75] |
| Top 10 PCs Combo | 0.83 [0.50, 1.00] | 0.64 [0.11, 1.00] | **0.85 [0.75, 0.92]** | **0.85 [0.76, 0.92]** | 0.86 [0.70, 0.97] | 0.84 [0.72, 0.94] | 0.80 [0.64, 0.94] | **0.83 [0.70, 0.92]** |
| Concatenated PCs | 0.56 [0.39, 1.00] | 0.26 [0.03, 0.92] | 0.71 [0.58, 0.82] | 0.69 [0.57, 0.80] | 0.57 [0.37, 0.72] | 0.81 [0.67, 0.93] | 0.70 [0.48, 0.87] | 0.63 [0.45, 0.76] |

### Table S19. APOE4 classification: SBF vs. ICA comparators

**Table S19. Model performance in predicting *APOE4* status, comparing SBF loadings–based models, demographic comparator model, and ICA comparator models.**

| **Model** | **AUROC (test)** | **AUPRC (test)** | **Accuracy (CV)** | **Balanced Accuracy (CV)** | **Sensitivity (CV)** | **Specificity (CV)** | **Precision (CV)** | **F1 (CV)** |
| --- | --- | --- | --- | --- | --- | --- | --- | --- |
| SBF Loadings | 0.82 [0.67, 0.93] | 0.75 [0.52, 0.94] | 0.70 [0.64, 0.77] | **0.67 [0.61, 0.74]** | 0.53 [0.42, 0.64] | 0.82 [0.75, 0.88] | 0.66 [0.54, 0.77] | **0.58 [0.49, 0.68]** |
| Demographics | 0.53 [0.36, 0.70] | 0.42 [0.27, 0.62] | 0.61 [0.54, 0.68] | 0.52 [0.50, 0.54] | 0.04 [0.00, 0.09] | **0.99 [0.97, 1.00]** | **0.75 [0.00, 1.00]** | 0.07 [0.00, 0.16] |
| Amyloid ICs | **0.89 [0.75, 0.98]** | 0.86 [0.67, 0.98] | **0.71 [0.64, 0.77]** | **0.67 [0.60, 0.73]** | 0.45 [0.32, 0.55] | 0.89 [0.83, 0.94] | 0.72 [0.58, 0.84] | 0.55 [0.43, 0.65] |
| Tau ICs | 0.57 [0.25, 0.63] | 0.63 [0.41, 0.79] | 0.63 [0.55, 0.68] | 0.60 [0.53, 0.66] | 0.49 [0.37, 0.58] | 0.72 [0.63, 0.79] | 0.54 [0.40, 0.66] | 0.51 [0.40, 0.60] |
| Grey Matter ICs | 0.47 [0.30, 0.65] | 0.46 [0.29, 0.67] | 0.65 [0.59, 0.72] | 0.59 [0.53, 0.64] | 0.26 [0.17, 0.36] | 0.91 [0.86, 0.96] | 0.67 [0.48, 0.83] | 0.38 [0.25, 0.49] |
| Cortical Thickness ICs | 0.51 [0.31, 0.66] | 0.48 [0.29, 0.68] | 0.61 [0.53, 0.67] | 0.60 [0.53, 0.67] | 0.58 [0.47, 0.69] | 0.62 [0.53, 0.70] | 0.51 [0.40, 0.62] | 0.54 [0.45, 0.63] |
| Pial Surface Area ICs | 0.52 [0.31, 0.65] | 0.44 [0.27, 0.64] | 0.52 [0.45, 0.59] | 0.53 [0.46, 0.60] | 0.58 [0.47, 0.69] | 0.48 [0.38, 0.57] | 0.43 [0.34, 0.53] | 0.49 [0.40, 0.58] |
| Top 10 ICs Combo | 0.52 [0.29, 0.68] | 0.51 [0.29, 0.78] | 0.44 [0.37, 0.51] | 0.52 [0.48, 0.56] | **0.95 [0.89, 0.99]** | 0.10 [0.04, 0.15] | 0.41 [0.34, 0.48] | 0.57 [0.50, 0.64] |
| Concatenated ICs | 0.88 [0.76, 0.97] | **0.87 [0.73, 0.97]** | **0.71 [0.64, 0.77]** | 0.66 [0.60, 0.72] | 0.42 [0.33, 0.53] | 0.89 [0.84, 0.95] | 0.73 [0.59, 0.85] | 0.53 [0.43, 0.63] |

Values are shown with 95% confidence intervals on the second line of each cell. The best performance under each metric is bolded. CV means the result was from cross-validated training data.

# I. Component 28 Validation: Amyloid Quantification and *APOE4* Carrier Status

To independently validate the interpretation of Component 28 as an amyloid-driven pattern, we examined its relationship to regional amyloid quantification using PET Centiloid measures. Regional Centiloid values were derived from standardized uptake value ratios (SUVRs) using established tracer-specific linear calibrations, and aggregated into lobar composites as surface-area-weighted means across bilateral cortical parcellations, with subcortical regions weighted by regional volume. The Component 28 Centiloid was computed as the mean amyloid PET uptake weighted by the Component 28 spatial map, and compared with these regional Centiloid composites across diagnostic groups and *APOE4* carrier status.

As shown in Figure S8, Component 28 Centiloid (panel A) increased across diagnostic stages (CN → MCI → dementia) for both *APOE4* carriers and non-carriers, closely paralleling standard occipital Centiloid measures (panel B). *APOE4* carriers exhibited higher values at earlier stages (CN and MCI), whereas non-carriers showed a more pronounced increase from MCI to dementia, consistent with a significant Diagnosis × *APOE4* interaction (Component 28 Centiloid: p = 0.017; occipital Centiloid: p = 0.006). This pattern indicates that Component 28 captures a biologically meaningful, regionally specific amyloid signal that varies with both disease stage and genotype.


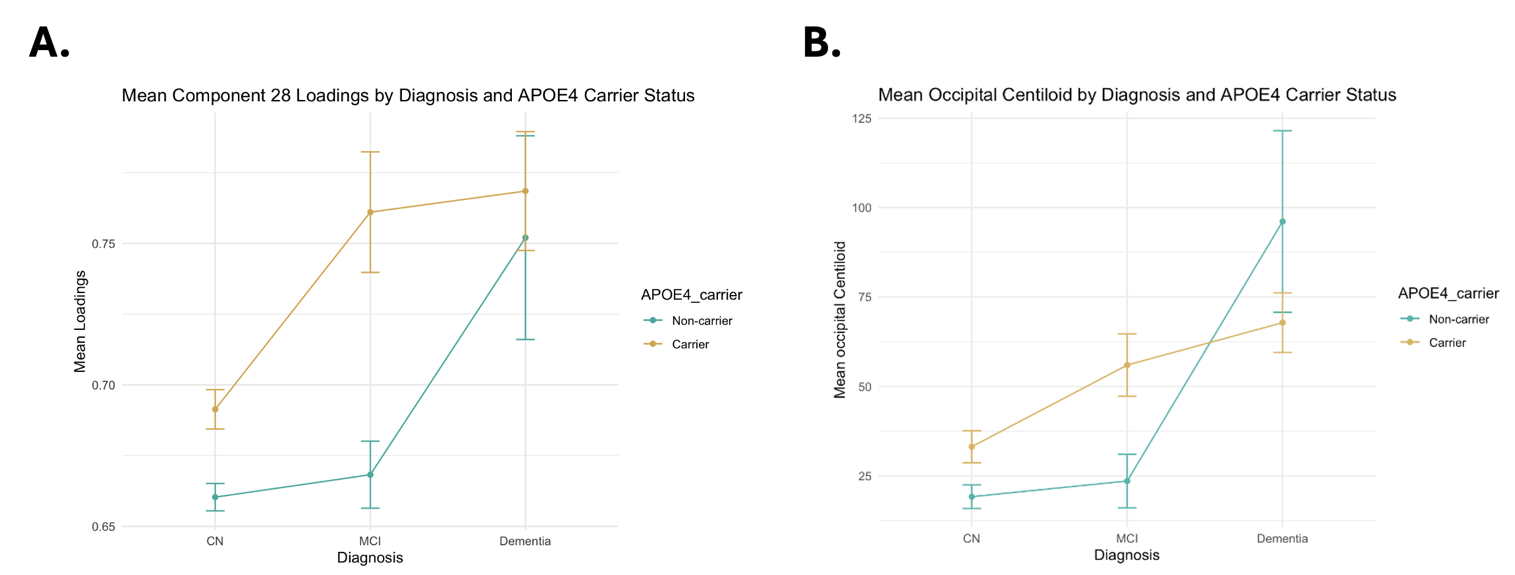


### Figure S8. Component Centiloid measures by diagnosis and APOE4 status

**Figure S8.** *Component loadings and Centiloid measures by diagnosis and APOE4 carrier status.* (A) Mean Component 28 loadings across diagnostic groups, stratified by *APOE4* carrier status. (B) Mean occipital Centiloid from standard regional amyloid quantification. Error bars indicate 95% confidence intervals.

# J. Age Confound Sensitivity Analysis

PCA-based cortical thickness features showed consistently strong performance across diagnostic classification tasks (Supplement E), raising the question of whether some of this predictive power reflects age-related variance rather than disease-specific signal. Cortical thinning is associated with both normal aging and neurodegeneration, and since age correlates with diagnostic group membership, features encoding age information could inflate classification performance in ways unrelated to disease biology. We address this with two complementary analyses using the same pre-defined train/test holdout as the main analysis.

First, we tested whether the feature loadings encode age information by training LASSO regression models to predict chronological age from SBF and PCA loadings. PCA LASSO predicted age consistently (r = 0.54), while SBF LASSO showed near-zero age prediction (r = 0.02–0.19). However, per-feature age R² for CT PCs was low (median < 1%, maximum 7–11% across comparisons), indicating that the age signal is distributed across many components rather than concentrated in any individual PC.

Second, we directly tested whether age-encoded variance in CT PCs contributes to classification. Age was regressed out of all feature loadings using a linear model fitted on training subjects only and applied to both train and test sets, and the same one-vs-one classification pipeline was re-run on the residualized features. CT PCA AUC was unchanged for the two comparisons where it performed most strongly: CN vs. Mild Dementia (0.949 → 0.949) and MCI vs. Mild Dementia (0.917 → 0.917). A small reduction was observed for MCI vs. CN (0.718 → 0.670, ΔAUC = −0.048), within bootstrap confidence intervals and comparable in magnitude to changes seen in other modalities. SBF performance was stable or slightly improved across all three comparisons.

Together, these results indicate that CT PCA’s classification advantage is not primarily driven by age-related variance. Although PCA loadings encode aggregate age information (detectable via multivariate LASSO), this signal is distributed across many components and does not meaningfully contribute to diagnosis classification. The dementia-relevant discriminative signal in CT PCs appears distinct from age-related cortical atrophy. SBF achieves comparable CDR‑SOB predictive performance without encoding age, consistent with its disease-specific design.


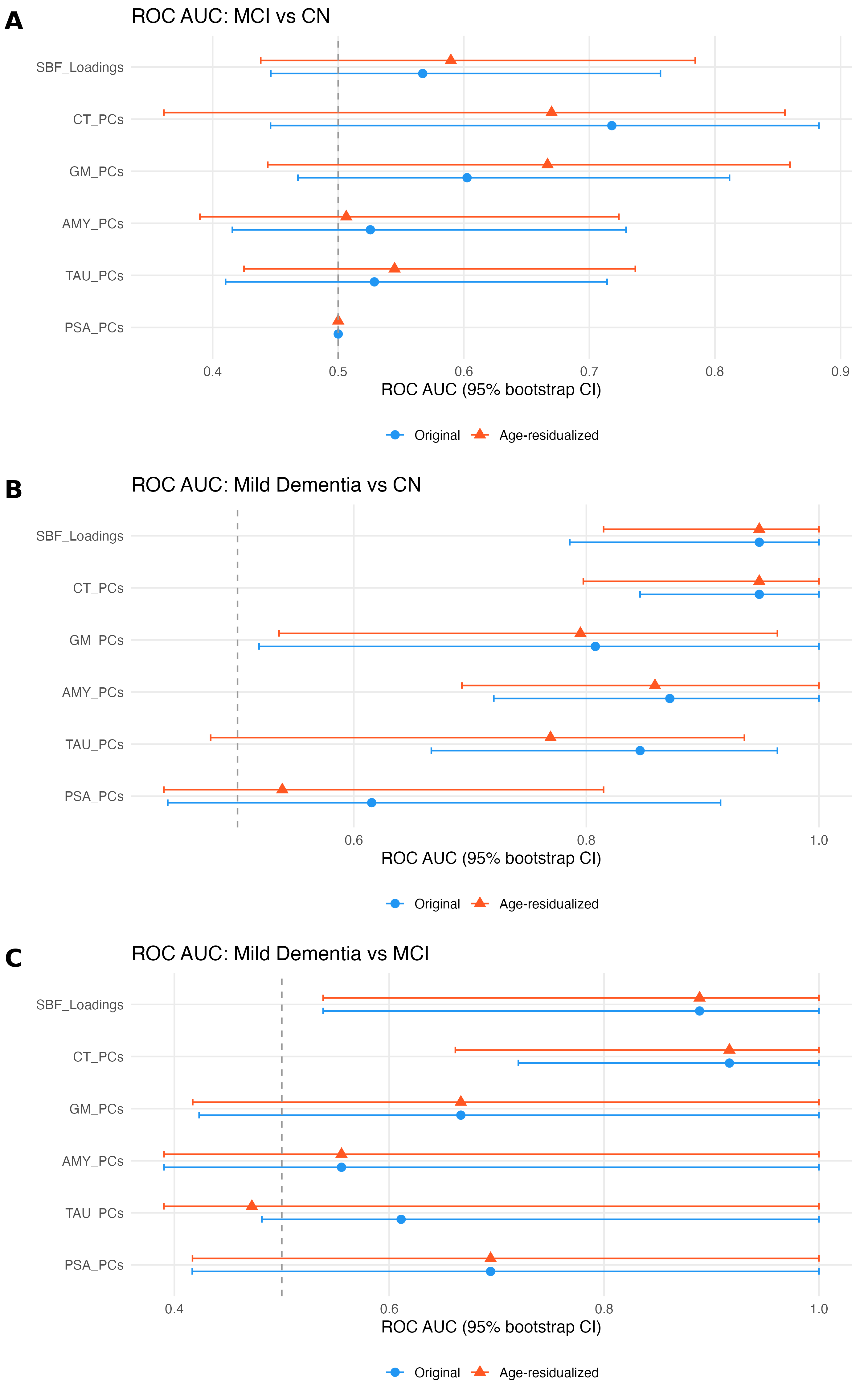


### Figure S9. Age residualization sensitivity analysis

Figure S9. Age residualization sensitivity analysis. ROC AUC (with 95% bootstrap CI) for each model before (blue circles, Original) and after (orange triangles, Age-residualized) regressing age out of feature loadings, shown for all three one-vs-one diagnostic comparisons: A. MCI vs. CN, B. Mild Dementia vs. CN, C. Mild Dementia vs. MCI. Age residualization was fitted on training subjects only and applied to both train and test sets. For the two comparisons where CT PCs performed most strongly (B, C), AUC is unchanged after residualization. A small reduction is observed for MCI vs. CN (CT_PCs: 0.718 → 0.670), within bootstrap confidence intervals.
